# Integrative multi-omics analysis for identifying novel therapeutic targets and predicting immunotherapy efficacy in lung adenocarcinoma

**DOI:** 10.20517/cdr.2024.91

**Published:** 2025-01-14

**Authors:** Zilu Chen, Kun Mei, Foxing Tan, Yuheng Zhou, Haolin Du, Min Wang, Renjun Gu, Yan Huang

**Affiliations:** ^1^Nanjing University of Chinese Medicine, Nanjing 210023, Jiangsu, China.; ^2^Department of Cardiothoracic Surgery, The Third Affiliated Hospital of Soochow University, Changzhou 213003, Jiangsu, China.; ^3^School of Chinese Medicine and School of Integrated Chinese and Western Medicine, Nanjing University of Chinese Medicine, Nanjing 210023, Jiangsu, China.; ^4^Jinling Hospital, Affiliated Hospital of Medical School, Nanjing University, Nanjing 210046, Jiangsu, China.; ^5^Department of Ultrasound, Nanjing Hospital of Chinese Medicine Affiliated with Nanjing University of Chinese Medicine, Nanjing 210022, Jiangsu, China.; ^#^Authors contributed equally.

**Keywords:** Lung adenocarcinoma, tumor immune microenvironment, immunotherapy, molecular subtype, multiomics

## Abstract

**Aim:** Lung adenocarcinoma (LUAD), the most prevalent subtype of non-small cell lung cancer (NSCLC), presents significant clinical challenges due to its high mortality and limited therapeutic options. The molecular heterogeneity and the development of therapeutic resistance further complicate treatment, underscoring the need for a more comprehensive understanding of its cellular and molecular characteristics. This study sought to delineate novel cellular subpopulations and molecular subtypes of LUAD, identify critical biomarkers, and explore potential therapeutic targets to enhance treatment efficacy and patient prognosis.

**Methods:** An integrative multi-omics approach was employed to incorporate single-cell RNA sequencing (scRNA-seq), bulk transcriptomic analysis, and genome-wide association study (GWAS) data from multiple LUAD patient cohorts. Advanced computational approaches, including Bayesian deconvolution and machine learning algorithms, were used to comprehensively characterize the tumor microenvironment, classify LUAD subtypes, and develop a robust prognostic model.

**Results:** Our analysis identified eleven distinct cellular subpopulations within LUAD, with epithelial cells predominating and exhibiting high mutation frequencies in Tumor Protein 53 (*TP53)* and Titin (*TTN)* genes. Two molecular subtypes of LUAD [consensus subtype (CS)1 and CS2] were identified, each showing distinct immune landscapes and clinical outcomes. The CS2 subtype, characterized by increased immune cell infiltration, demonstrated a more favorable prognosis and higher sensitivity to immunotherapy. Furthermore, a multi-omics-driven machine learning signature (MOMLS) identified ribonucleotide reductase M1 (RRM1) as a critical biomarker associated with chemotherapy response. Based on this model, several potential therapeutic agents targeting different subtypes were proposed.

**Conclusion:** This study presents a comprehensive multi-omics framework for understanding the molecular complexity of LUAD, providing insights into cellular heterogeneity, molecular subtypes, and potential therapeutic targets. Differential sensitivity to immunotherapy among various cellular subpopulations was identified, paving the way for future immunotherapy-focused research.

## INTRODUCTION

Lung adenocarcinoma (LUAD), the most prevalent subtype of non-small cell lung cancer (NSCLC), remains a significant health burden due to its high mortality and limited treatment options^[1]^. Despite advances in early detection and therapeutic interventions, patient prognosis remains poor, underscoring the urgent need for novel diagnostic and therapeutic strategies. Approximately 80% of patients with LUAD are diagnosed at advanced stages, missing the window for optimal treatment and significantly reducing their survival prospects^[2]^. Although targeted therapies and immunotherapies have provided new treatment options, resistance and relapse remain significant challenges^[3-6]^.

One of the significant hurdles in treating LUAD is the development of drug resistance, which arises from various mechanisms, including genetic mutations, epigenetic alterations, and changes in the tumor microenvironment (TME)^[7-9]^. Mutations in tumor protein 53 (*TP53*) and Titin (*TTN*) genes play pivotal roles in LUAD, influencing tumor aggressiveness and prognosis, and the patient’s response to treatments, including chemotherapy and immunotherapy^[10,11]^. These mutations disrupt cell cycle regulation, apoptosis, and DNA repair, making cancer cells more resistant to conventional therapies. Furthermore, the TME, characterized by immune evasion and the presence of cancer stem cells, significantly contributes to resistance against immunotherapy. Understanding these mechanisms is vital for the development of more effective treatment strategies.

The advent of high-throughput sequencing technologies, particularly single-cell RNA sequencing (scRNA-seq), has revolutionized our understanding of the cellular and molecular landscapes of cancers, including LUAD^[12]^. By profiling gene expression at the single-cell level, researchers can identify distinct cellular subpopulations and elucidate their roles in tumor initiation, progression, immune evasion, and therapeutic resistance. Recent studies have leveraged scRNA-seq to identify novel cellular subpopulations across various cancers, underscoring their critical roles in tumor progression and treatment response^[13-15]^. Moreover, investigations into intrinsic resistance mechanisms at immune checkpoint blockade (ICB) sites through comprehensive single-cell profiling of melanoma have shown that targeting transcription factor 4 (*TCF4)* expression increases sensitivity to ICBs and targeted therapies^[16]^.

This study conducted an integrative bioinformatics analysis, combining scRNA-seq, bulk transcriptomic, and genome-wide association study (GWAS) data from multiple LUAD cohorts. The study objective was to identify LUAD-associated cellular subpopulations, characterize their functional roles, and elucidate the molecular mechanisms driving LUAD pathogenesis. Recent multi-omics analyses have significantly advanced our understanding of cancer biology, offering more profound insights into tumor heterogeneity and identifying potential therapeutic targets^[17,18]^.

Our analysis revealed nine cellular subpopulations in LUAD, with epithelial cells being the most prevalent. Through Bayesian deconvolution, the functional landscapes of these subpopulations were mapped; essential genes and pathways associated with LUAD were identified. A machine-learning-based prognostic model was developed, which demonstrated robust predictive power for patient outcomes. Furthermore, the TME and immune landscape of LUAD were examined, identifying significant variations in immune cell infiltration and immune-related gene expression across the molecular subtypes. Many of these genes have been associated with resistance to immunotherapy. Our findings suggest potential therapeutic targets and strategies for improving the efficacy of immunotherapy in patients with LUAD.

In summary, this study provides a comprehensive multi-omics approach to dissecting the molecular complexity of LUAD, focusing on drug resistance. It offers valuable insights into cellular heterogeneity, molecular subtypes, and potential therapeutic targets, establishing a foundation for future research to improve the diagnosis, prognosis, and treatment of LUAD.

## METHODS

### Data retrieval and preprocessing

This study included nine research cohorts: one single-cell dataset (GSE189357), seven transcriptomic datasets [The Cancer Genome Atlas (TCGA)-LUAD, GSE31210, GSE50081, GSE78220, GSE91061, GSE135222, IMvigor210] and one GWAS dataset (ieu-a-984). Further details of these cohorts are available in Supplementary Materials.

### Single-cell sequencing analysis

Quality control, batch effect correction, dimensionality reduction, clustering, and cell annotation were conducted to identify nine cellular subpopulations and predict intercellular communication. More details are provided in Supplementary Materials.

### Bayesian deconvolution for cell type and gene expression inference

Using the scPagwas package^[19]^, disease-associated cellular subpopulations were identified at the single-cell level, trait-related scores (TRS) were calculated, and trait-related genes were pin-pointed. Deconvolution analysis was applied to determine the score contribution of each cellular subpopulation to transcriptomic samples. Additional details are included in Supplementary Materials.

### Identification of functional differences in complex cellular subpopulations

Differential gene expression, immune function variability, response to immunotherapy, drug prediction, and somatic mutations within specific complex cellular subpopulations were identified. Subsequently, hub module genes in these subpopulations were identified using the Weighted Gene Co-expression Network Analysis (*WGCNA*) package. Further details are provided in Supplementary Materials.

### Multi-omics integration analysis

Multi-omics data, including mRNA, lncRNA, miRNA, and methylation profiles, were integrated from the TCGA-LUAD cohort. Through multi-omics consensus clustering analysis using the Modular View of Cancer Subtypes (*MOVICS)* package, distinct subtypes were identified. Supplementary Materials contains additional information.

### Molecular profiling via consensus clustering

To assess the characteristics of LUAD subtypes and their association with various treatment features, Gene Set Variation Analysis (GSVA) was employed. Further details are provided in Supplementary Materials.

### Construction of prognostic models using machine learning

A machine learning-based predictive model was developed; its predictive value was assessed using Kaplan-Meier survival curves. A nomogram was constructed to enhance its clinical relevance. More detailed methods can be found in Supplementary Materials.

### Immunogenomic profiling and response to immunotherapy

Leveraging the multi-omics-driven machine learning signature (MOMLS), TME cell types, immunotherapy response, immune suppression, and immune rejection characteristics were analyzed in high and low MOMLS score groups using the Immune-Oncology Biomarker Resource (*IOBR)* package. Supplementary Materials provides additional details.

### Screening of potential therapeutic agents for patients with a MOMLS profile

Gene Set Enrichment Analysis (GSEA) algorithm and Profile Reporting Information System and Management (PRISM) data were used to predict drug sensitivity for those with MOMLS profiles. More details are available in Supplementary Materials.

### Colocalization analysis

Colocalization analysis used the Colocalization (*COLOC*) package to mitigate the risk of confounding between exposure and outcome due to related but distinct causal variants. Additional methodological details are available in Supplementary Materials.

### Statistical analysis

All statistical and bioinformatics analyses were performed using R software. Continuous variables were compared using *t*-tests or Mann-Whitney tests, depending on the data distribution. Kaplan-Meier curves and Cox regression analyses were used to assess differences in clinical outcomes. Multi-omics integration was prepared using the *MOVICS* package. Statistical significance was set at a *P*-value of < 0.05.

## RESULTS

### scRNA-seq identifies key cellular landscape in LUAD

**Figure 1:**
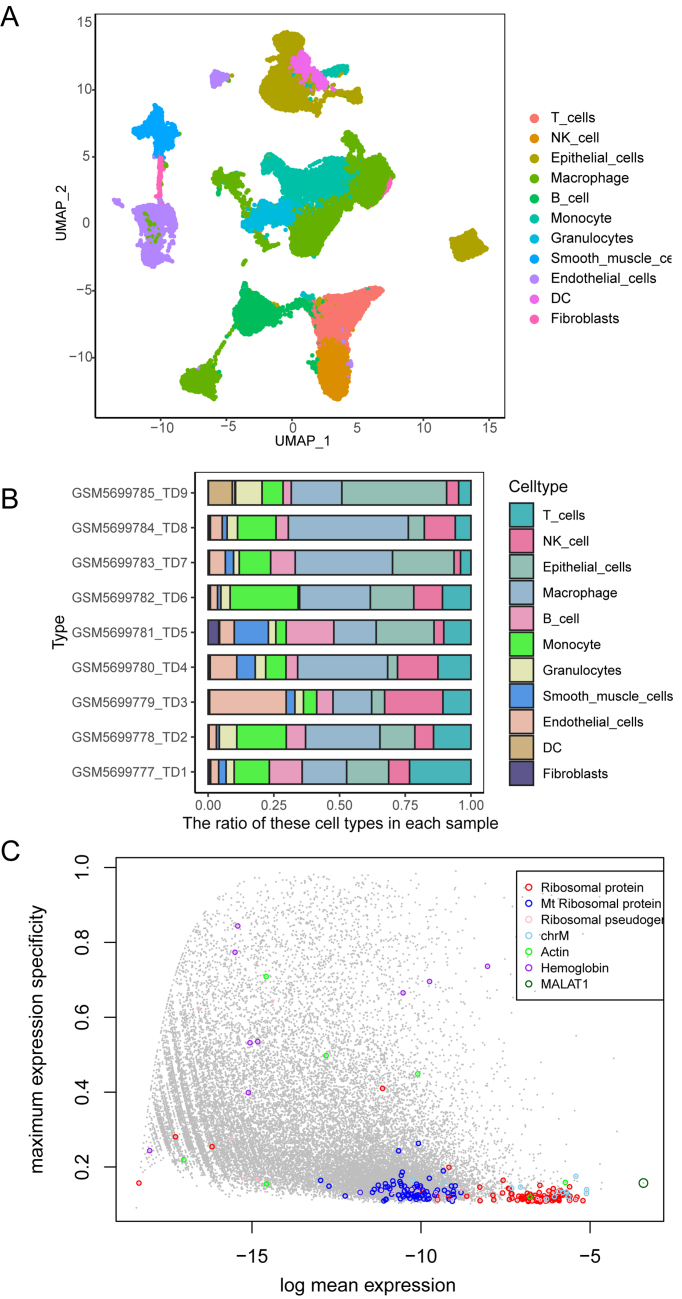
Single-cell RNA sequencing data reveal the trait-associated cellular LUAD. (A) UMAP plot illustrating eleven distinct LUAD cell types; (B) Proportions of the nine LUAD cell types; (C) Average gene expression and cell type specificity scores. LUAD: Landscape of lung adenocarcinoma; UMAP: Uniform Manifold Approximation and Projection; DC: dendritic cells. ^*^P < 0.05; ^**^P < 0.01; ^***^P < 0.001; ^****^P < 0.0001.

The scRNA-seq data was analyzed from 48,814 cells obtained from nine LUAD samples after quality control. Cell annotation using the singleR package and Manual operation created a comprehensive atlas of LUAD, identifying eleven distinct cell types [Figure 1A]. Quantification of these cell types across samples [Figure 1B] showed that epithelial cells were the most dominant population. Correlation analysis revealed that epithelial cells strongly correlated with dendritic cells (DC) [Supplementary Figure 1A]. Consequently, epithelial cells were selected for Bayesian deconvolution in LUAD. Ribosomal protein and mitochondrial genes were excluded from this analysis to avoid biases due to their high distributional dominance and lack of cell type-specificity. These genes typically exhibit elevated expression with minimal specificity [Figure 1C], making them unsuitable for accurate deconvolution.

**Figure 2:**
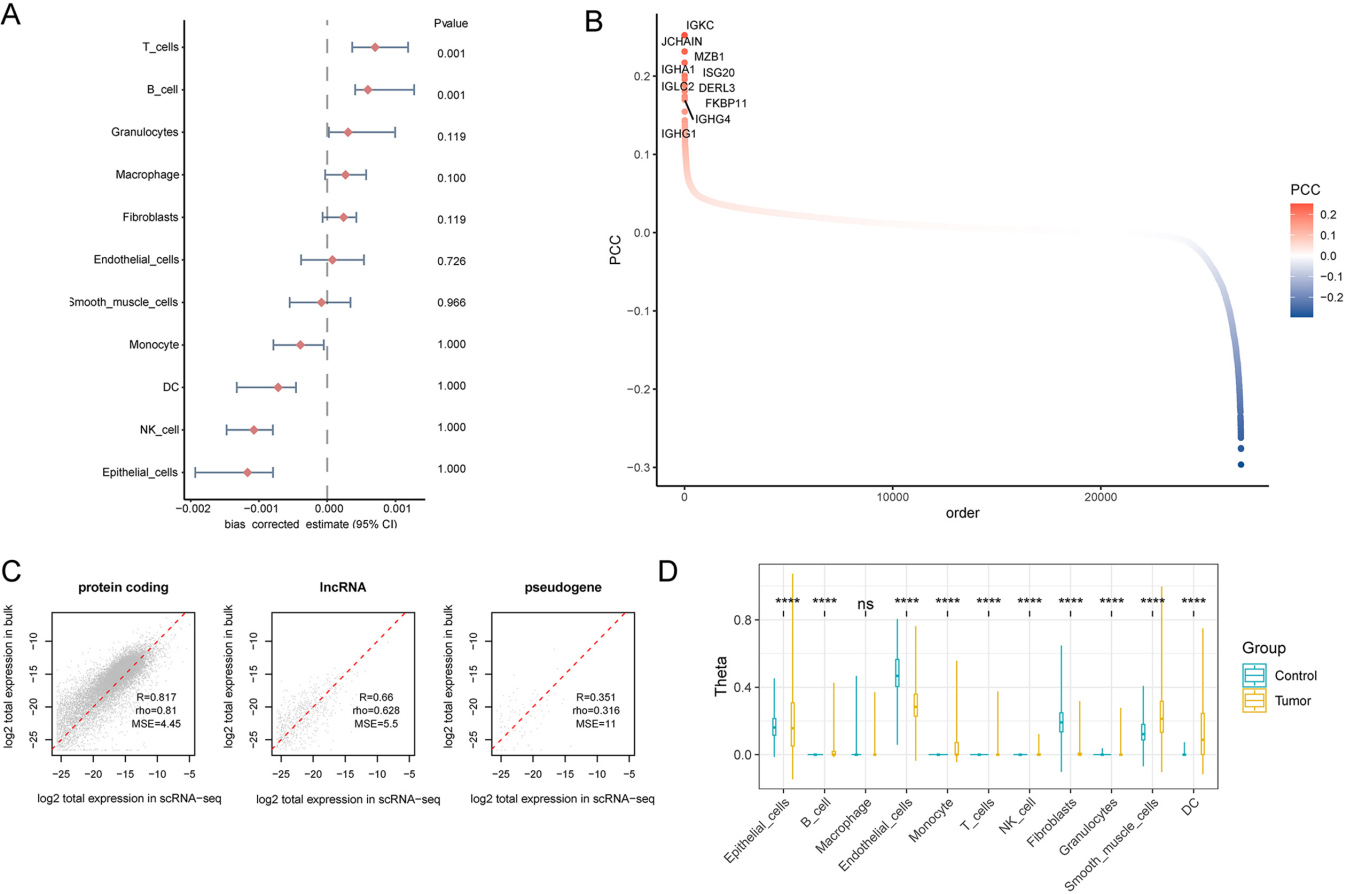
Bayesian deconvolution analysis of single-cell RNA sequencing data. (A) Correction of scPagwas cell subpopulations; (B) Ranking of trait-associated genes for each cell by scPagwas; (C) Bayesian deconvolution analysis results; (D) Differential transcriptome analysis across cell subpopulations. DC: Dendritic cells; PCC : pearson correlation coefficient. ^*^P < 0.05; ^**^P < 0.01; ^***^P < 0.001; ^****^P < 0.0001.

LUAD epithelial cell types were integrated with LUAD GWAS summary data (11,245 cases and 54,619 controls) using the scPagwas package, calculating the overall TRS for epithelial cells. These scores differed significantly from other cell types [Supplementary Figure 1B], although this difference diminished after statistical correction [Figure 2A]. Furthermore, the top 1,000 trait-associated genes in LUAD epithelial cells were identified by calculating correlations between the expression of each given gene and the epithelial cell types, visualizing the top 10 trait genes [Figure 2B]. Differential analysis on TCGA-LUAD bulk-transcriptome data further identified differentially expressed genes [Supplementary Figure 1C and D]. Convolution analysis revealed a strong correlation between single-cell epithelial data and protein-coding genes (R = 0.817) from TCGA-LUAD [Figure 2C]. Subpopulation scores were computed for each sample [Supplementary Table 1].

### Functional analysis of epithelial cells in LUAD

Further analysis of epithelial cell subpopulations in TCGA-LUAD revealed significant differences in convoluted epithelial cells [Figure 2D]. Differential analysis identified various upregulated genes; the top five are visualized in a volcano plot [Supplementary Figure 2A]. Patients in TCGA-LUAD were divided into high- and low-score groups based on epithelial convolution cell (ECC) scores. Estimation of Stromal and Immune cells in Malignant Tumors using Expression data (ESTIMATE*)* immune scoring and tumor purity analysis revealed significant differences between these groups [Supplementary Figure 2B], suggesting that epithelial cells play critical roles in tumor immunity, progression, and resistance to treatment.

**Figure 3:**
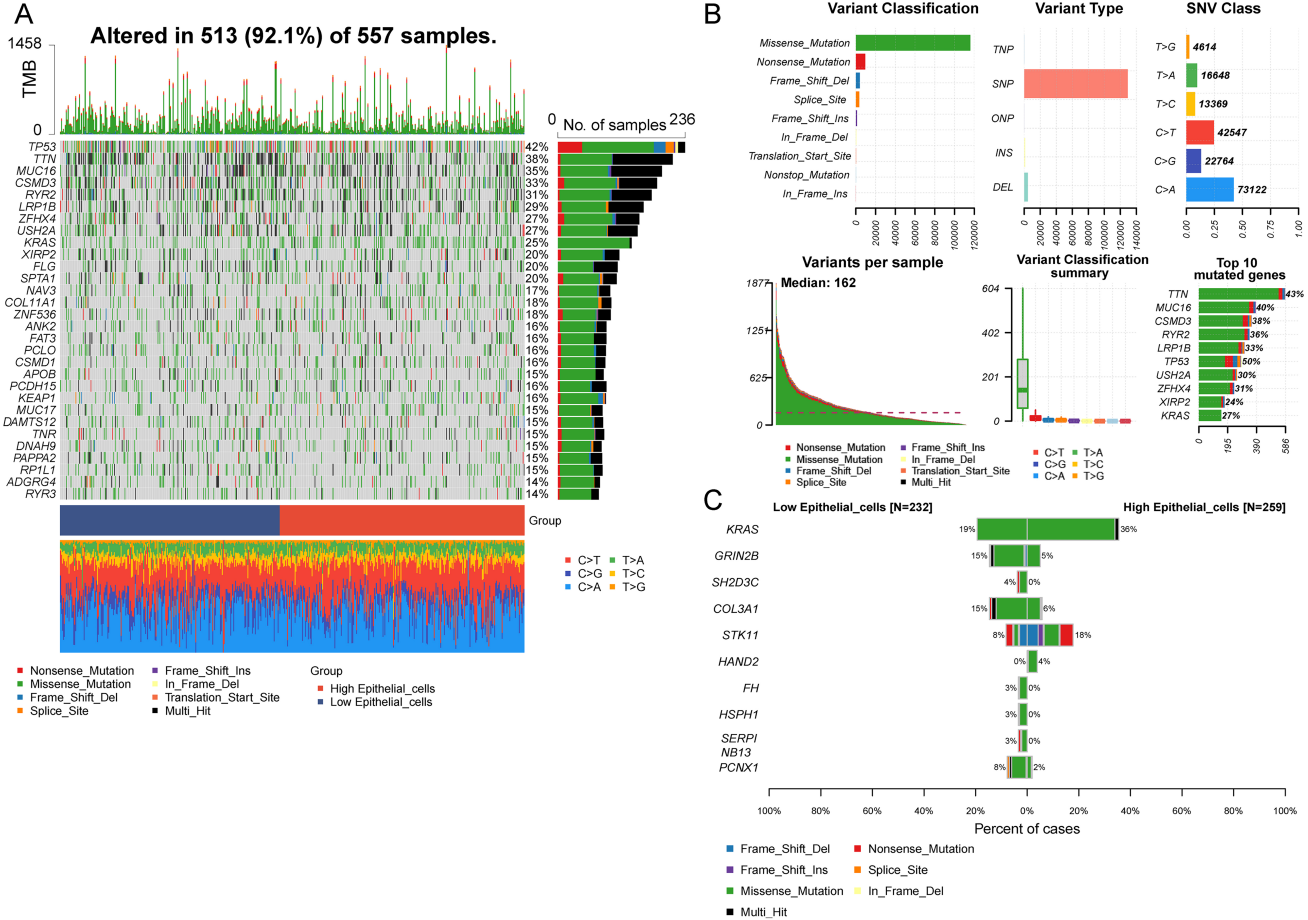
Mutation landscape in epithelial convolution cells of LUAD. (A) Waterfall plot illustrating somatic mutations in epithelial convolution cells of LUAD; (B) Types of somatic mutations in these cells. The meanings of colors in the figure are annotated in (A); (C) Differentially mutated somatic genes in epithelial convolution cells of LUAD. LUAD: Landscape of lung adenocarcinoma; TMB : tumor mutation burden ; SNV : single nucleotide variants.

The high ECC score group had a greater mutation frequency than the low-score group for somatic mutations. The most frequent mutations in the high-score group were in *TP53* (42%), *TTN* (38%), and Mucin 16 (*MUC16)* (35%) genes [Figure 3A], with missense mutations being the most prevalent [Figure 3B]. Kirsten Rat Sarcoma Viral Oncogene Homolog (*KRAS)* mutation rates differed substantially between the two groups, with a 36% mutation rate in the high ECC score group *vs.* 19% in the low-score group [Figure 3C]. Differentially mutated genes between these groups are displayed in Supplementary Figure 3.

Given the critical role of immunotherapy and tumor immunity in LUAD progression, various immune gene sets were examined within ECCs. Natural killer cell cytotoxicity, antigen processing and presentation, chemokine receptors, interleukins, interferons, and their receptors were observed in the low-score group, suggesting an activated immune state that enhances tumor recognition and clearance [Supplementary Figure 4].

### Identification of key module genes in epithelial cells

**Figure 4:**
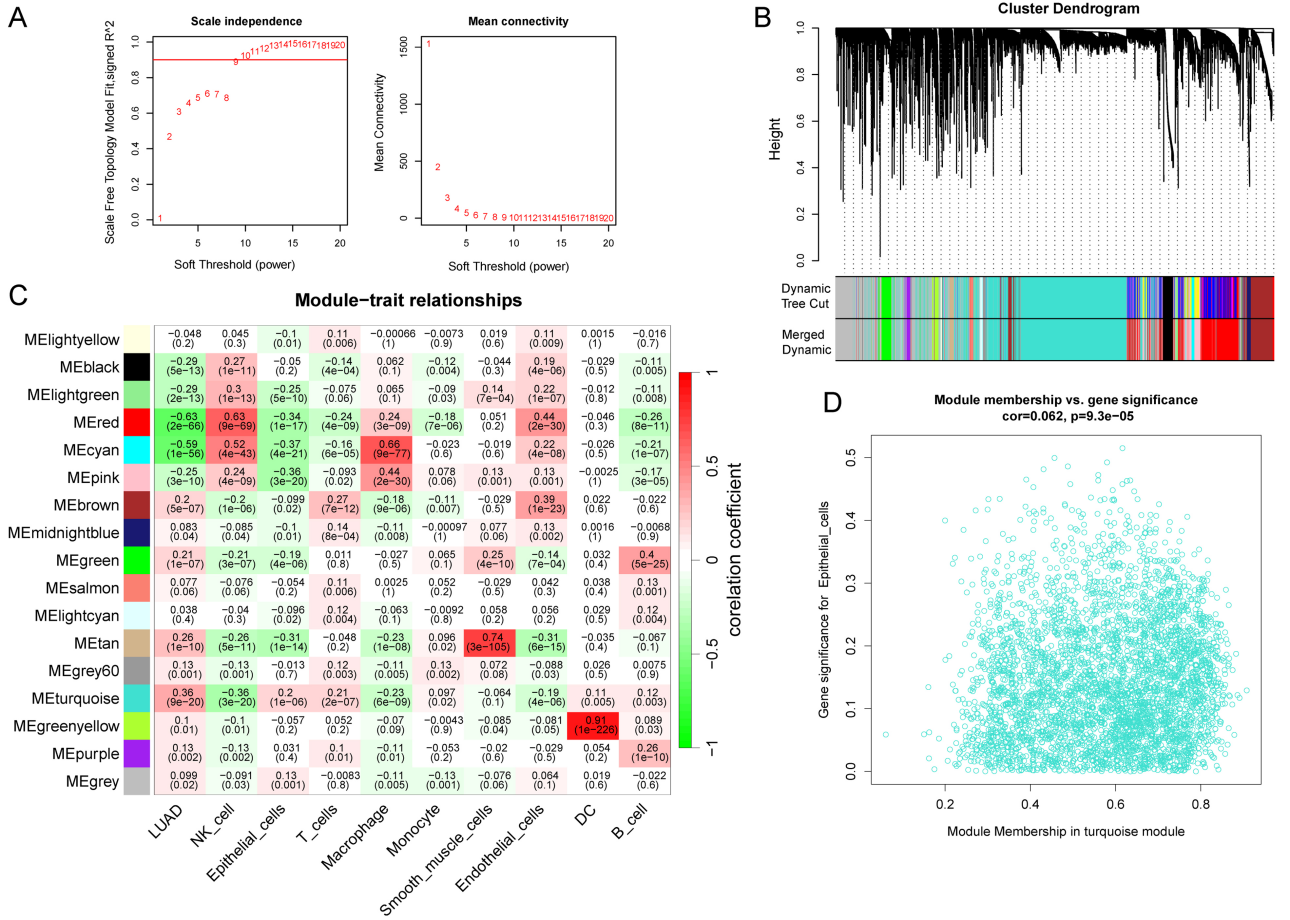
Identification of WGCNA hub modules in epithelial convolution cells of LUAD. (A) Analysis of scale-free topology index and mean connectivity analysis for various soft threshold powers; (B) Identification of co-expression gene modules: the dendrogram branches into 17 distinct modules, each represented by a unique color; (C) Heatmap showing the correlation between modules and trait gene sets, including gene significance and corresponding *P*-values; (D) Scatter plot illustrating the correlation between module membership and gene significance for the turquoise module. WGCNA: Weighted Gene Co-expression Network Analysis; LUAD: landscape of lung adenocarcinoma; DC: dendritic cells.

Critical genes within ECCs of LUAD were identified using the WGCNA package. A scale-free topology network was constructed by selecting an optimal soft-threshold power of nine, and 17 gene modules were identified using a minimum size of 50 genes each [Figure 4A and B]. The turquoise module was most significantly associated with epithelial cell scores [Figure 4C]. A scatter plot of gene significance (GS) and module membership (MM) in the turquoise module confirmed this substantial correlation [Figure 4D], suggesting that genes within this module may have functional importance in epithelial cells.

### Identification of immune subtypes in LUAD using multi-omics data

**Figure 5:**
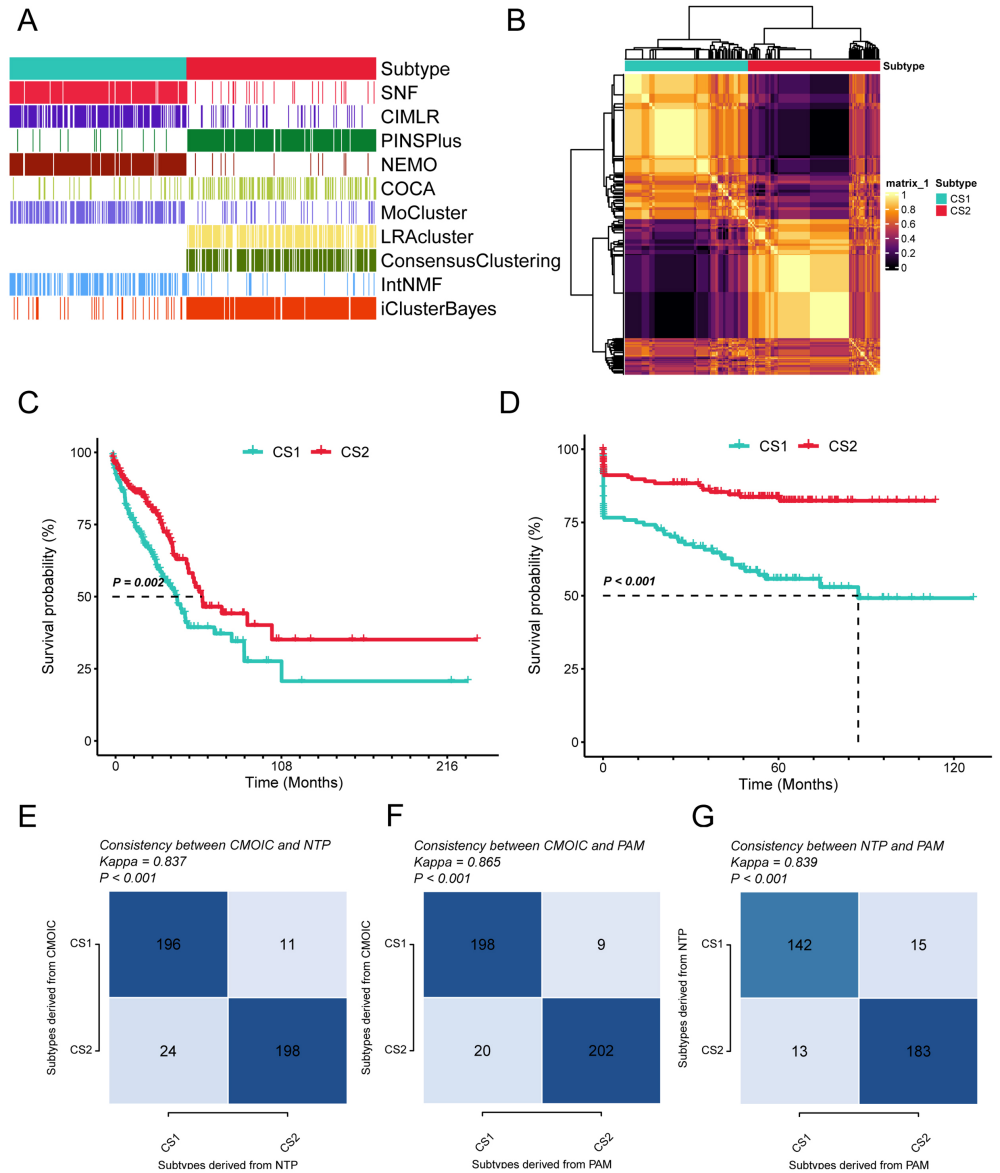
Multi-omics identification of immune subtypes in LUAD. (A) Multi-omics clustering approach for LUAD; (B) Consensus clustering matrix of two prognostic subtypes based on ten algorithms; (C) KM curves comparing the two subtypes; (D) KM curves for subtypes in the meta cohort; (E) Consistency analysis between CS and NTP in the TCGA-LUAD cohort; (F) Consistency between CS and PAM in the TCGA-LUAD cohort; (G) Consistency between NTP and PAM in the meta-LUAD cohort. LUAD: Landscape of lung adenocarcinoma; KM: Kaplan-Meier; CS: consensus subtype; TCGA: The Cancer Genome Atlas; PAM: partitioning around medoids; CIMLR: Cancer Integration via Multikernel Learning; SNF: Similarity Network Fusion; PINSPlus: Perturbation Clustering for data INtegration Plus; NEMO: Network-based Ensemble Method for Omics data; COCA: Consensus Clustering with multiple Algorithms; moCluster: multiple omics cluster; LRACluster: low-rank approximation cluster; IntNMF: integrative non-negative matrix factorization; NTP: Nearest Template Prediction.

Immune subtypes significantly influence the effectiveness of immunotherapy in lung cancer. This study used transcriptomic, DNA methylation, and somatic mutation data to classify LUAD into subtypes. Two LUAD subtypes were identified using ten multi-omics clustering algorithms [consensus subtype (CS)1 and CS2, Figure 5A and B, Supplementary Figure 5A]. Survival analysis revealed a significant difference in prognosis between the subtypes (*P* = 0.002), with CS2 demonstrating a more favorable long-term outcome than CS1 [Figure 5C]. The molecular characteristics distinguishing these subtypes were examined, focusing on cancer chromatin remodeling regulators and transcription factors [Supplementary Figure 5B]. Specifically, *FGFR3, RXRA, GATA6*, and *AR* were significantly enriched in CS2, while *FOXM1* and *HIF1A* were prominent in CS1. These findings suggest that epigenetic regulation plays a pivotal role in the molecular divergence of LUAD subtypes. Furthermore, immune cell infiltration was observed, observing greater levels of B and T cells in CS2 [Supplementary Figure 5C]. Validation using a dataset confirmed the robustness of the identified subtypes, with CS2 exhibiting superior survival rates [*P* < 0.001, Figure 5D and Supplementary Figure 5D]. Finally, the classification accuracy was validated using the Nucleotide Transition Probability (NTP) algorithm, supporting our conclusions [Figure 5E-G].

### Construction of prognostic model MOMLS

**Figure 6:**
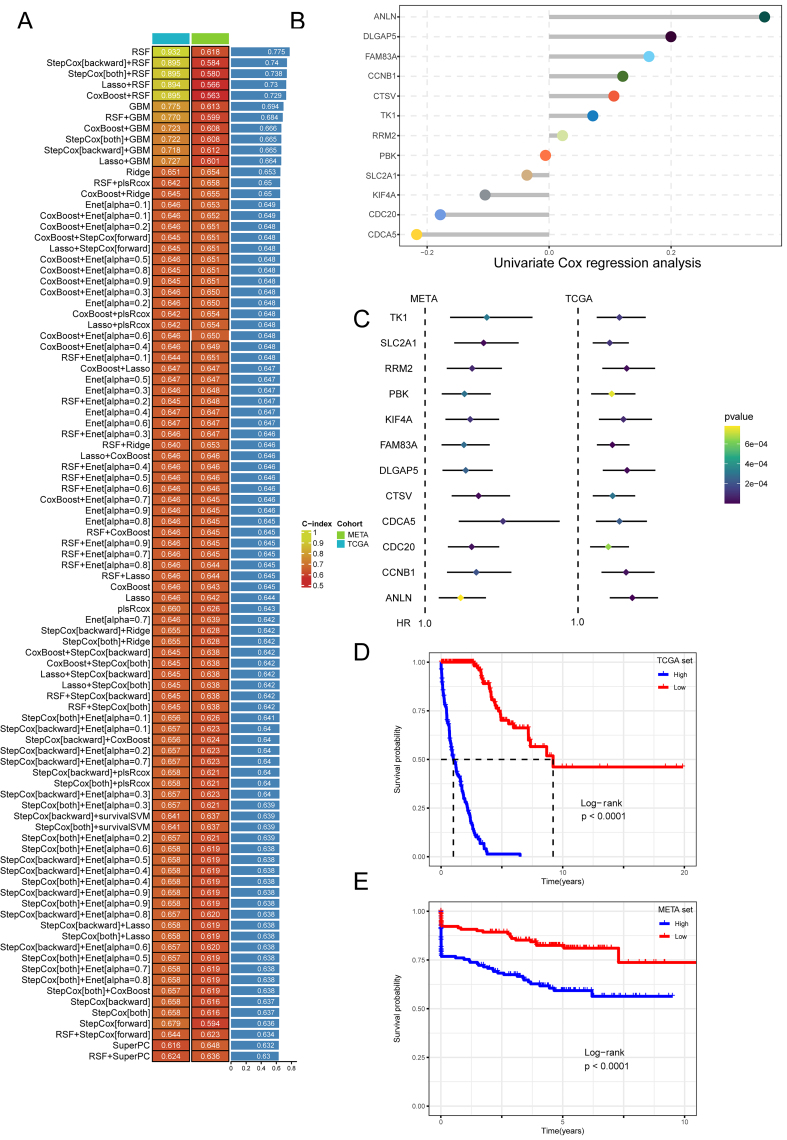
Machine learning construction of the prognostic model MOMLS. (A) Each model’s concordance index (C-index) derived from 101 machine-learning algorithms in the TCGA-LUAD and meta cohorts, ranked by the average C-index from the validation set; (B) The gene framework for the model constructed using the RSF algorithm. Each color represents each gene; (C) Univariate Cox regression analysis results for hub genes in the training and validation cohorts; (D and E) KM curves for the high and low MOMLS score groups in the meta-LUAD and meta cohorts. MOMLS: Multi-omics-driven machine learning signature; TCGA: The Cancer Genome Atlas; LUAD: landscape of lung adenocarcinoma; RSF: random survival forest; KM: Kaplan-Meier; Enet: elastic net; GBM: generalized boosted regression model; SuperPC: supervised principal components; plsRcox: partial least Cox.

**Figure 7:**
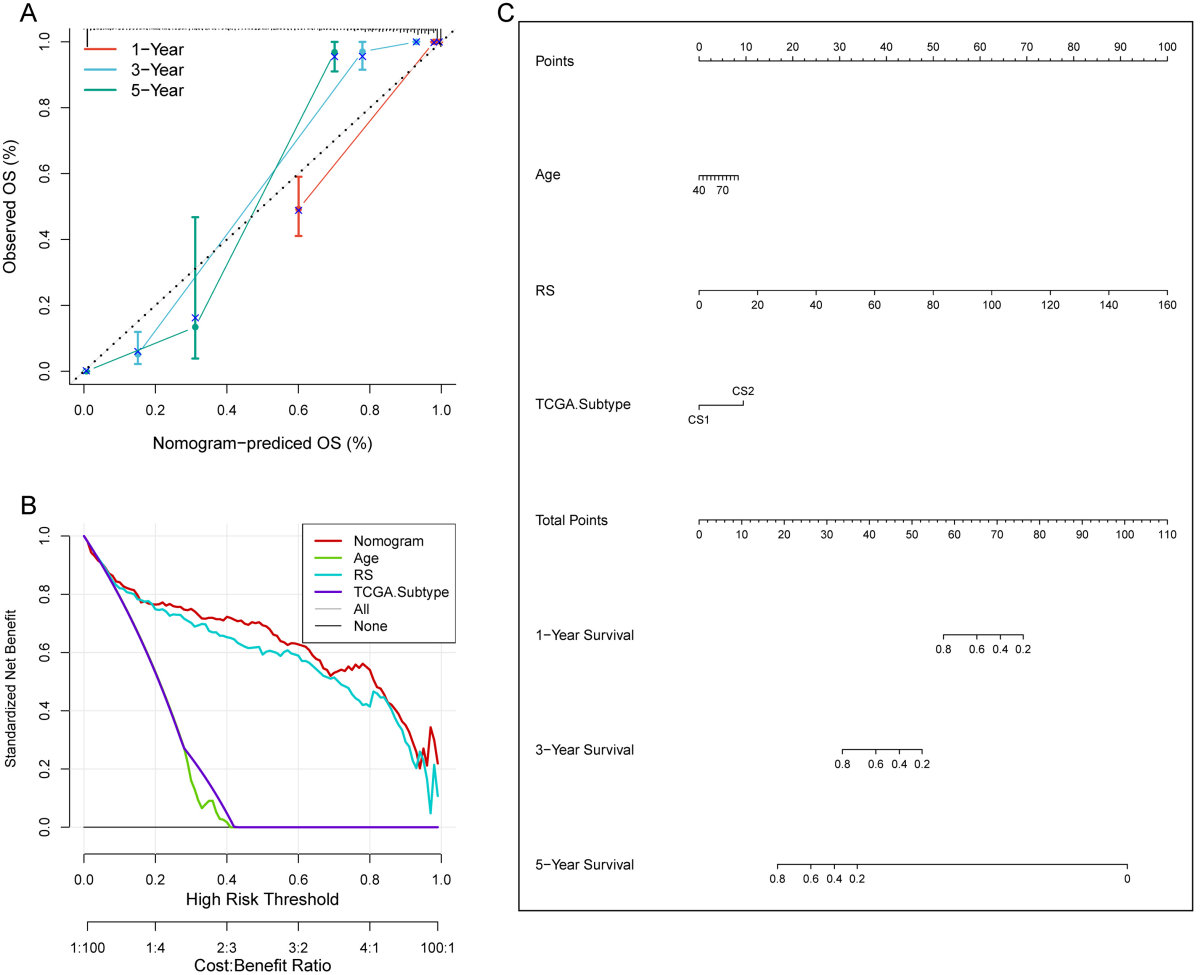
Model evaluation of prognostic model MOMLS. (A) Comprehensive clinical nomogram based on MOMLS; (B) The 1, 3, and 5-year forecast curves of the nomogram; (C) Analysis of the decision curve in the nomogram. MOMLS: Multi-omics-driven machine learning signature; TCGA: The Cancer Genome Atlas; RS: risk score.

A total of 101 combinations of machine learning algorithms were created utilizing 30 screened hub genes to identify the optimal model construction method. This analysis incorporated TCGA-LUAD and meta-datasets, evaluating each model’s predictive capability using the average concordance index (C-index). The Random Survival Forest (RSF) algorithm produced the highest average C-index [Figure 6A], leading us to develop a prognostic model based on RSF-included genes [Figure 6B] and compute the Cox regression index for model genes across both validation and training sets [Figure 6C]. The prognostic evaluation indicated that cohorts with low MOMLS scores had improved survival outcomes in training and validation datasets [*P* < 0.0001, Figure 6D and E]. Comparative analysis revealed that MOMLS outperformed other models^[20-41]^ in C-index performance [Supplementary Figure 6A and B]. A nomogram incorporating relevant prognostic factors was constructed for clinical applicability, which demonstrated excellent calibration with actual outcomes, outperforming MOMLS alone in decision-making utility [Figure 7].

### Immune characteristics of MOMLS

**Figure 8:**
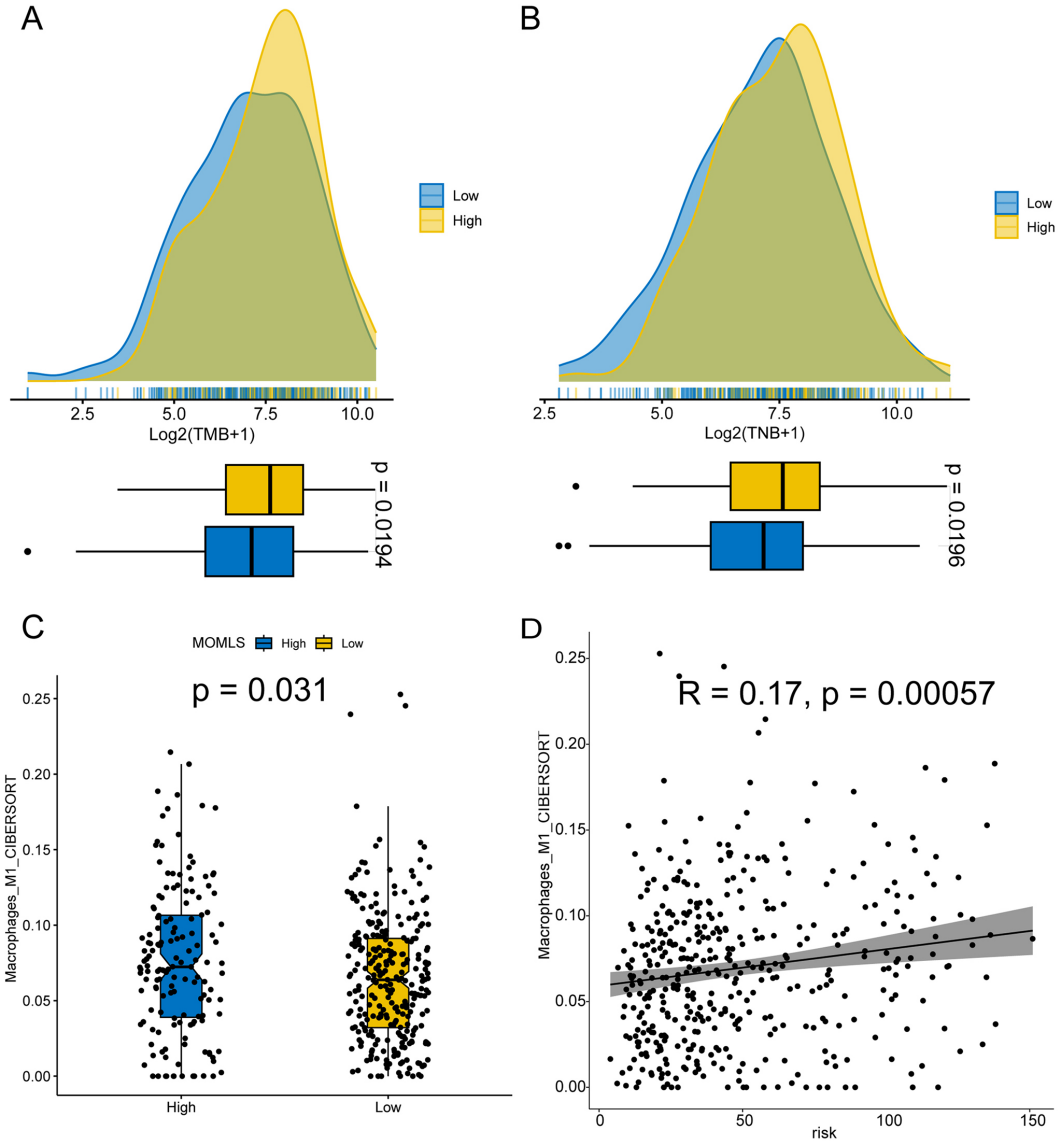
Immune Characteristics of MOMLS. (A) TMB distribution in those with high and low MOMLS scores; (B) TNB in those with high and low MOMLS scores; (C) Differences in M1 macrophage levels between the high and low MOMLS score groups; (D) Scatter plot illustrating the relationship between MOMLS and M1 macrophage levels. MOMLS: Multi-omics-driven machine learning signature; TMB: tumor mutational burden; TNB: tumor neoantigen burden.

A comprehensive analysis of the TME related to LUAD-associated MOMLS was conducted, revealing significantly higher immune cell levels in the low MOMLS score group compared to the high-score group, indicative of an activated immune state [Supplementary Figure 7]. This finding suggests that patients with low MOMLS scores are more likely to exhibit "hot tumors," which may contribute to differences in drug resistance to immunotherapy. Tumor mutational burden (TMB) and tumor neoantigen burden (TNB) are recognized biomarkers for evaluating patient response to immunotherapy. Higher TMB, TNB, and M1 macrophage enrichment were observed in the low-score cohort, suggesting enhanced immunogenicity [Figures 8]. Survival analysis indicated that MOMLS could effectively differentiate patient prognoses based on TMB, TNB, and M1 macrophage levels [Supplementary Figure 6C-E], with patients exhibiting low MOMLS scores showing improved survival rates when combined with high TMB, TNB, or M1 macrophage infiltration.

### Immune therapy response and drug treatment prediction for MOMLS

**Figure 9:**
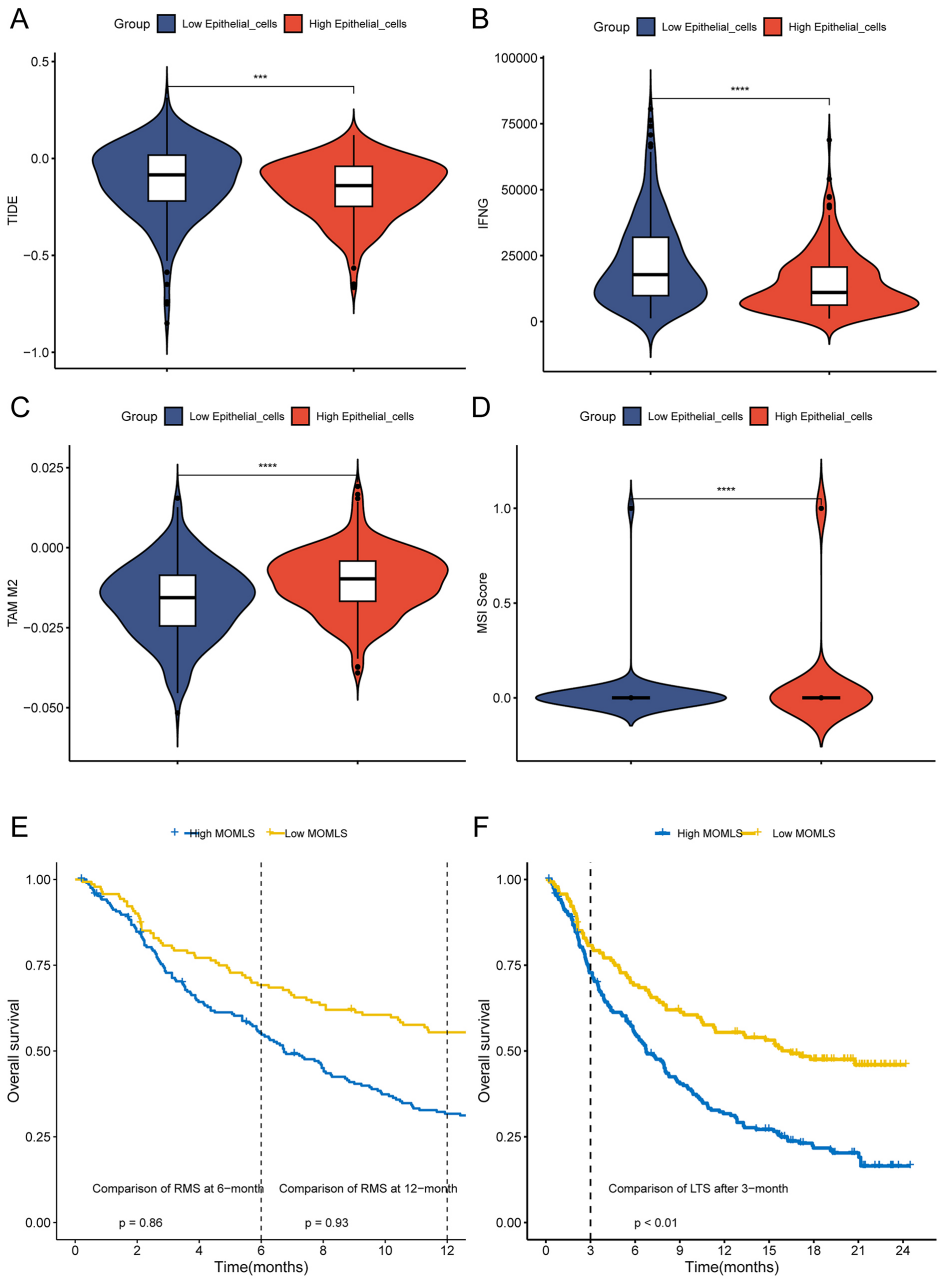
Immune landscape of MOMLS. (A-D) TIDE immune therapy analysis for epithelial convolution cells; (E) Differences in RMS after 6 months and one year of treatment between high and low MOMLS score groups; (F) LTS differences after 3 months of treatment between high and low MOMLS score groups. MOMLS: Multi-omics-driven machine learning signature; TIDE: tumor immune dysfunction and exclusion; RMS: restricted mean survival time; LTS: long-term survival. ^*^P < 0.05; ^**^P < 0.01; ^***^P < 0.001; ^****^P < 0.0001.

**Figure 10:**
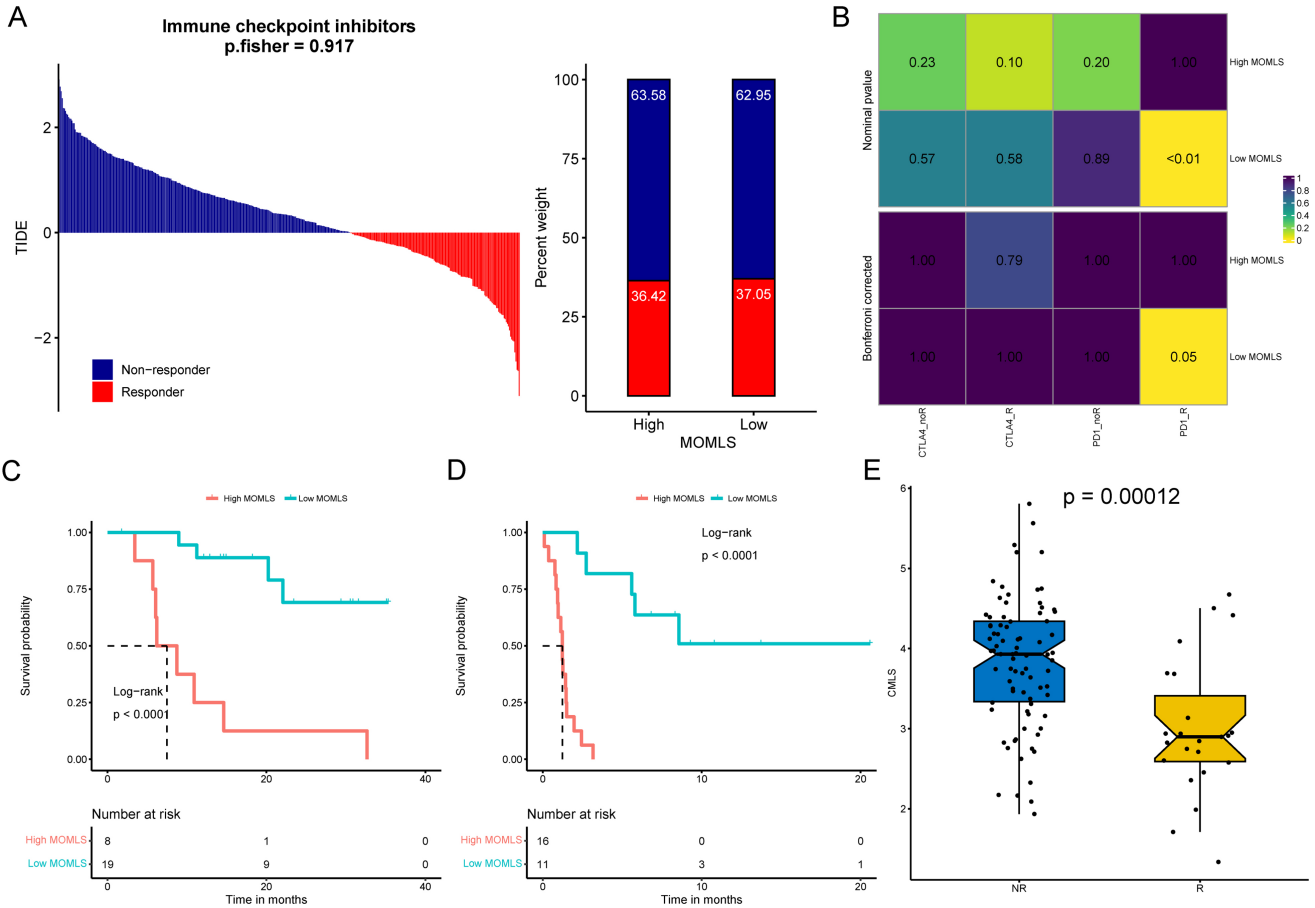
Immunotherapy response and survival analysis of MOMLS. (A) Distribution of MOMLS across various immune therapy response groups; (B) TIDE algorithm predictions of immune therapy responses for high and low MOMLS groups; (C) Subclass mapping algorithm predictions of immune therapy responses between high and low MOMLS groups; (D) KM curves for high and low MOMLS groups in the GSE78220 immune dataset; (E) Distribution of MOMLS in different immune therapy datasets, including GSE91061. MOMLS: Multi-omics-driven machine learning signature; TIDE: tumor immune dysfunction and exclusion; KM: Kaplan-Meier; CTLA-4: cytotoxic T-lymphocyte-associated protein 4; PD-1: programmed cell death protein 1.

A Tumor Immune Dysfunction and Exclusion (TIDE) analysis focusing on ECCs was initially conducted to assess the response to immunotherapy in MOMLS. The results indicated that the group with low ECC score had a higher likelihood of tumor immune evasion and reduced efficacy of immune checkpoint inhibitors (ICIs) [Figure 9A-D and Supplementary Figures 8A-F]. Although the IMvigor210 cohort showed no significant differences in restricted mean survival (RMS) at 6 and 12 months between the high and low MOMLS score groups, patients in the low-score group had a better prognosis after 3 months of treatment [Figure 9E and F]. Furthermore, those who responded to treatment exhibited improved outcomes compared to non-responders [Supplementary Figure 9]. The TIDE algorithm revealed no significant variation in immunotherapy response between MOMLS score groups [Figure 10A], contrasting with our ECC calculations. However, validation using an independent immunotherapy dataset demonstrated a more robust response to programmed cell death protein 1 (PD-1) inhibitors in the low MOMLS score group [Figure 10B]. Consistently, across multiple immunotherapy validation cohorts, patients in the low MOMLS score group showed better prognosis following treatment [Figure 10C and D] and improved immunotherapy outcomes [Figure 10E].

**Figure 11:**
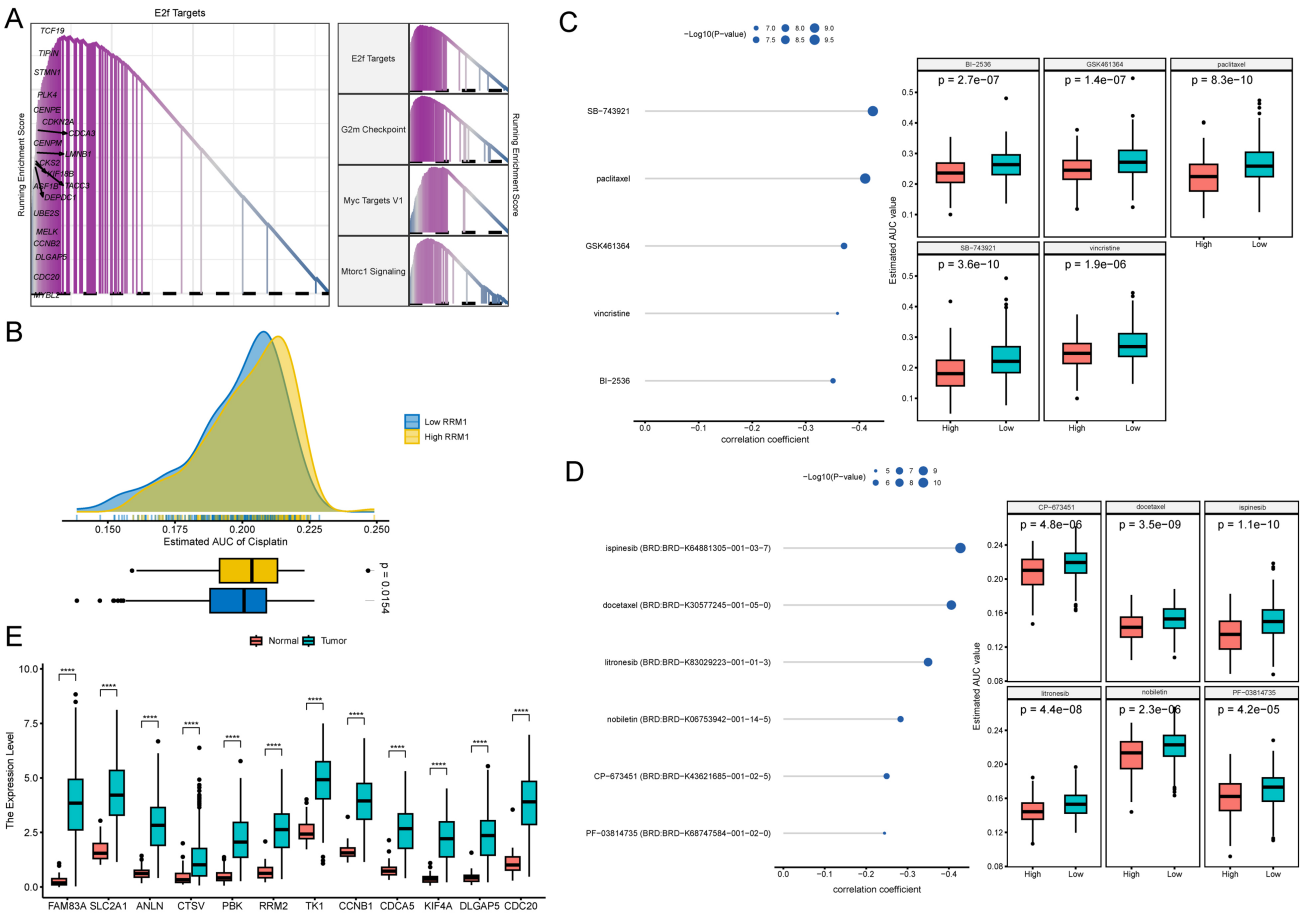
Drug prediction of MOMLS. (A) Pathways significantly activated in the high MOMLS score group as identified by the GSEA algorithm; (B) Predicted sensitivity of *RRM1*; (C-D) Correlation and differential analyses of drug sensitivity for potential drugs screened from the CTRP and PRISM datasets; (E) Differential expression analysis of hub genes in normal *vs.* tumor tissues. MOMLS: Multi-omics-driven machine learning signature; GSEA: Gene Set Enrichment Analysis; *RRM1:* Ribonucleotide reductase M1; CTRP: Cancer Therapeutics Response Portal; PRISM: Profile Reporting Information System and Management; AUC: area under curve. ^*^P < 0.05; ^**^P < 0.01; ^***^P<0.001; ^****^P<0.0001.

GSEA indicated pathways such as E2 promoter-binding factor (E2F) targets were significantly activated in patients with high MOMLS scores [Figure 11A]. Ribonucleotide reductase M1 (RRM1), a rate-limiting enzyme involved in ribonucleotide reduction, plays a crucial role in DNA synthesis and repair. Elevated *RRM1* expression in lung cell lines leads to Phosphatase and Tensin Homolog (*PTEN*) upregulation, which inhibits cell migration, invasion, and metastasis. In transgenic mouse models, high RRM1 levels reduce the formation of carcinogen-induced lung tumors and are associated with gemcitabine resistance. Low *RRM1* expression in tumors correlates with better survival and improved outcomes for patients receiving gemcitabine and platinum-based therapies. Our results predict that patients with high *RRM1* expression will exhibit a favorable response to gemcitabine and platinum-based treatment, potentially improving chemotherapeutic outcomes [Figure 11B]. Potential therapeutic agents were screened for patients with high MOMLS scores, identifying drugs such as paclitaxel, docetaxel, and vincristine as potentially beneficial [Figure 11C and D, Supplementary Figure 8G]. Gene target expression was compared in tumors, normal tissues, and ECCs [Figure 11E and Supplementary Figure 10].

### Colocalization analysis

Gene-trait colocalization analysis was performed to validate scPagwas and MOMLS prognostic genes. While no significant posterior probability was found between the analyzed genes and traits [Supplementary Table 2], likely due to the selected GWAS data, two genes, SECIS Binding Protein 2-Like (*SECISBP2L*) and Myelin Protein Zero-Like 3 (*MPZL3*), emerged as potential biomarkers associated with LUAD. These findings warrant further investigation in future studies.

## DISCUSSION

LUAD is a prevalent type of lung cancer with a high metastatic potential^[42]^. Identifying novel biomarkers and developing a comprehensive understanding of LUAD progression mechanisms are essential for improving therapeutic strategies^[43]^. Our study utilized scRNA-seq and multi-omics integration to elucidate the complex cellular and molecular landscape of LUAD. Nine distinct cellular subpopulations were identified, with epithelial cells being the most prominent. The intense interaction between epithelial cells and DCs suggests a significant interaction between the TME and immune response. Bayesian deconvolution revealed critical disease-associated genes in epithelial cells, with the high ECC score showing elevated mutation frequencies in *TP53*, *TTN*, and *MUC16*. Malignant epithelial cells are pivotal in tumor progression, contributing to chemotherapy resistance and immune evasion^[44-46]^. Our analysis revealed that high ECC score groups exhibit higher mutation frequencies in critical oncogenes such as *TP53, TTN*, and *MUC16*, previously linked to aggressive tumor behavior and resistance to conventional therapies^[47-49]^. One of the most commonly mutated genes in LUAD is *TP53*. It is a critical tumor suppressor regulating cell cycle arrest and apoptosis. Mutations in *TP53* compromise these regulatory processes, leading to uncontrolled cell proliferation and tumor growth^[50]^. *TTN*, while traditionally associated with muscle integrity, has recently gained attention in cancer biology. Mutations in *TTN* often occur in regions of high structural disorder, promoting genomic instability and aberrant cellular responses^[51]^. In contrast, *MUC16* is predominantly associated with promoting cancer cell survival and metastasis by engaging oncogenic pathways that drive tumor progression and increase chemoresistance^[52]^. Collectively, these mutations contribute to more aggressive disease phenotypes. The significant correlation between epithelial cells and DCs highlights a potential mechanism in TME modulation, promoting immune evasion and facilitating disease progression.

Furthermore, the enrichment of immune-related gene sets in the low ECC score group indicates an active immune environment, potentially more responsive to immunotherapy. Reporting diseases with different molecular subtypes is of great significance in influencing disease heterogeneity and molecular role in shaping diseases^[53,54]^. Our multi-omics analysis identified two LUAD subtypes (CS1 and CS2) with distinct molecular characteristics and survival outcomes. Patients with the CS2 subtype had higher infiltration of B and T cells compared to CS1, correlating with significantly improved long-term prognosis. This observation aligns with the findings of Zhang *et al*., where the CS2 subtype showed the highest immune cell infiltration and immune checkpoint gene expression, rendering them more responsive to PD-1 therapy and sensitive to docetaxel and cisplatin^[55]^. Conversely, the CS1 subtype was more sensitive to paclitaxel. These results underscore the importance of the immune microenvironment in determining patient outcomes and suggest that enhancing immune cell infiltration could improve the prognosis for patients with LUAD.

Furthermore, TIDE analysis demonstrated that low ECC score groups have more potential for tumor immune escape and diminished efficacy for ICIs. This finding suggests that patients with low ECC scores may exhibit reduced responses to ICI therapy, highlighting the need for alternative therapeutic strategies for this group. These results align with earlier research showing that tumors with low immune cell infiltration and elevated markers of immune evasion typically have poor responses to ICI therapy^[56,57]^. For instance, tumors with low CD8+ T cell presence and high expression of inhibitory molecules such as programmed death-ligand 1 (PD-L1) and cytotoxic T-lymphocyte-associated protein 4 (CTLA-4) are more likely to escape immune surveillance and exhibit resistance to immunotherapy^[58,59]^. The CS2 subtype, with higher immune cell infiltration, shows better responses to immunotherapies, while the CS1 subtype, characterized by lower immune infiltration, aligns with increased immune evasion and reduced efficacy of ICIs. This subtype-specific immune behavior suggests that different therapeutic strategies are needed to effectively target these immune escape mechanisms and overcome drug resistance. This subtype-specific immune behavior suggests that different therapeutic strategies are needed to effectively target these immune escape mechanisms and overcome drug resistance. For instance, therapies focusing on enhancing T cell activation and infiltration may be more suitable for CS1, aiming to counteract its immune-suppressive microenvironment. On the other hand, the CS2 subtype, with its more robust immune activity, could benefit from combination therapies that not only maintain T cell response but also address potential adaptive resistance pathways. These findings highlight the importance of tailoring treatment plans according to the unique immune features of each subtype, ultimately improving patient outcomes and advancing personalized medicine in LUAD.

Our findings regarding RRM1 expression are particularly significant. Classical studies indicate that elevated RRM1 levels in lung cell lines enhance *PTEN* expression, leading to decreased cell migration, invasion, and metastasis^[60,61]^. Moreover, high RRM1 levels have been linked to inhibiting carcinogen-induced lung tumors in transgenic mice and are associated with gemcitabine resistance^[62,63]^. Clinical studies have reported that patients exhibiting low *RRM1* expression in tumor tissues experience improved survival and better therapeutic outcomes when treated with gemcitabine and platinum-based therapies^[64,65]^. Interestingly, our results contradict earlier studies, suggesting potential biases in our calculations or indicating a bidirectional effect. However, our results support the notion that *RRM1* expression could be a biomarker for predicting chemotherapy response and guiding treatment decisions in patients with LUAD.

As sequencing technologies have advanced, substantial amounts of multi-omics data have been generated, yet effectively harnessing these data remains challenging. The MOMLS score integrates multiple omics datasets, optimizing personalized immunotherapy strategies^[66-68]^. For patients with high MOMLS scores, our analysis identified several potential therapeutic agents, including paclitaxel, docetaxel, and vincristine, which may address the unique therapeutic benefits of those with high MOMLS scores compared to their counterparts. This identification lays the groundwork for personalized treatment strategies aimed at improving outcomes for this subgroup of patients with LUAD. Moreover, the MOMLS score can facilitate monitoring patient responses during treatment and enable timely adjustments to therapy regimens for optimal results^[69]^. Interestingly, our study also revealed that different immune cell subgroups exhibited varied sensitivities to immunotherapy. The roles of these genes in drug resistance may shape the immune landscape by modulating tumor cell survival and proliferation, thereby impacting the efficacy of immunotherapy. For instance, the overexpression of these genes could foster an immune evasion phenotype, rendering tumors less responsive to ICIs.

While our study provides valuable insights into the cellular and molecular landscape of LUAD, it has several limitations. Firstly, the single-cell data were derived from a relatively small sample size, which may limit the generalizability of our findings. Future studies should involve larger cohorts to validate and extend our results. Secondly, while potential therapeutic targets and prognostic biomarkers were identified, functional validation of these findings *in vitro* and *in vivo* is necessary to confirm their clinical relevance. Thirdly, our study primarily focused on epithelial cells, necessitating further investigation of other cell types within the TME to gain a more comprehensive understanding of LUAD biology.

Future research should aim to validate our findings in more extensive, independent cohorts to ensure robustness and applicability across diverse patient populations. Functional studies are needed to elucidate how identified genes and cellular subpopulations influence LUAD progression and treatment response. Additional *in vivo* and *in vitro* experiments will increase the accuracy of our conclusions. Most importantly, our model highlights variations in immunotherapy sensitivity among various cellular subpopulations, providing direct guidance for future immunotherapy targeting priorities. For instance, in the treatment of LUAD involving RRM1 immunosuppressants, tumors frequently develop resistance. Leveraging our findings to study epithelial cells that demonstrate greater sensitivity to immunotherapy could yield different therapeutic outcomes.

In conclusion, our study presents a comprehensive multi-omics framework that elucidates the molecular complexity of LUAD. This study provides valuable insights into cellular heterogeneity, molecular subtypes, and potential therapeutic targets. These findings establish a foundation for future research to improve the diagnosis, prognosis, and treatment of LUAD, ultimately promoting personalized medicine approaches for this challenging cancer.

## DECLARATIONS

### Availability of data and materials

The datasets presented in this study are available in online repositories. Additional information is available in the Supplementary Files.

### Copyright

© The Author(s) 2025.
