# Supplementary material for: Integrative multi-omics analysis for identifying novel therapeutic targets and predicting immunotherapy efficacy in lung adenocarcinoma
Source: Cancer Drug Resist. 2025 Jan 14;8:3. doi: 10.20517/cdr.2024.91 (PMC11810459; doi:10.20517/cdr.2024.91)
Supplement: Supplementary file 1 [file cdr-8-3-SupplementaryFiles.zip › cdr7091-SupplementaryFiles/Supplementary Figures/Figure Legends.pdf]

Figure legends

Supplementary Figure 1. Cell correlation of LUAD and difference analysis of TCGA-LUAD. (A) Correlation among the nine LUAD cell types; (B) Trait-related scores of cell subpopulations. (C, D) Volcano plot and heatmap depicting differential analysis for The Cancer Genome Atlas (TCGA)-LUAD.

Supplementary Figure 2. Differential Landscape of Epithelial Convolution Cells. (A) Differentially expressed genes in epithelial convolution cells; (B) ESTIMATE immune scores and tumor purity. *p<0.05, **p<0.01, ***p<0.001, ****p<0.0001.

Supplementary Figure 3. Differentially mutated genes in somatic mutations.

Supplementary Figure 4. Variations in multiple immune gene sets in epithelial convolution cells of LUAD. (A) Analysis of natural killer cell cytotoxicity; (B) Analysis of antigen processing and presentation; (C) Analysis of chemokine receptors; (D) Analysis of interleukins; (E) Analysis of interleukin receptors; (F) Analysis of interferons; (G) Analysis of interferon receptors.

Supplementary Figure 5. Heatmap of LUAD classification via uniform clustering**.** (A) Consensus clustering heatmap for transcriptome, DNA methylation sites, and mutation genes; (B) Heatmap of regulatory and transcription factors related to cancer chromatin remodeling; (C) Immune landscape from TCGA-LUAD; (D) Validation using the meta cohort.

Supplementary Figure 6. Evaluation of the MOMLS prediction model. (A and B) Comparison of MOMLS with 22 other published models; (C-E) Kaplan–Meier curves for MOMLS concerning TMB, TNB, and M1 macrophages.

Supplementary Figure 7. Immune cell levels associated with MOMLS. (A) Comparison of TME immune cell type characteristics between patients with high and low MOMLS scores; (B) Immune rejection characteristics in those with high versus low MOMLS scores; (C) Immune inhibition characteristics in those with high versus low MOMLS scores; (D) Immune therapy biomarkers in those with high versus low

MOMLS scores.

Supplementary Figure 8. Immune and drug prediction for convolution cells. (A-F) TIDE immune therapy analysis for epithelial convolution cells; (G) Drug prediction for epithelial convolution cells.

Supplementary Figure 9. The distribution of MOMLS in different immunotherapy response groups.

Supplementary Figure 10. Correlation scatter plot of normal and tumor tissues hub genes.
