# Supplementary material for: Integrative multi-omics analysis for identifying novel therapeutic targets and predicting immunotherapy efficacy in lung adenocarcinoma
Source: Cancer Drug Resist. 2025 Jan 14;8:3. doi: 10.20517/cdr.2024.91 (PMC11810459; doi:10.20517/cdr.2024.91)
Supplement: Supplementary file 1 [file cdr-8-3-SupplementaryFiles.zip › cdr7091-SupplementaryFiles/Supplementary Figures/Supplementary Figure 3.pdf]

High Epithelial_cells (n = 259) v/s Low Epithelial_cells (n = 232)

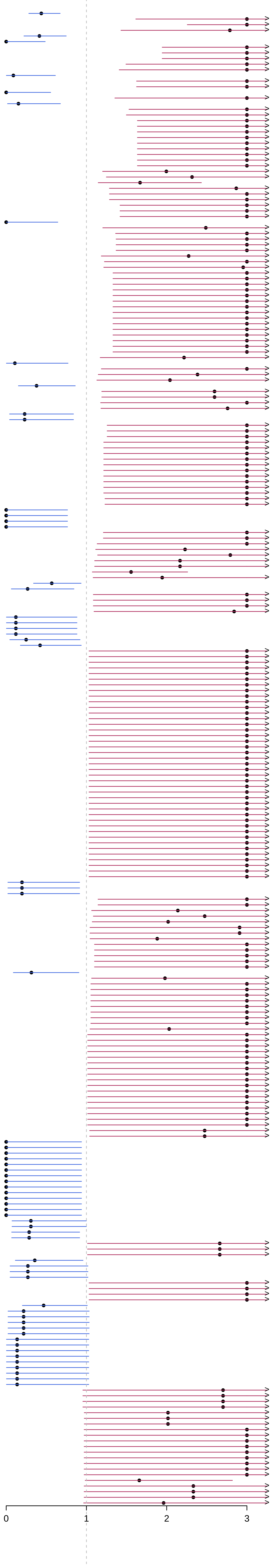

	High Epithelial_cells	Low Epithelial_cells	OR	P−value
KRAS	92	45	0.438	***
GRIN2B	13	34	3.242	***
SH2D3C	0	9	Inf	**
COL3A1	15	34	2.788	**
STK11	46	19	0.414	**
HAND2	10	0	0	**
FH	0	8	Inf	**
HSPH1	0	8	Inf	**
SERPINB13	0	8	Inf	**
PCNX1	5	18	4.261	**
ABCA10	6	19	3.752	**
NUTM1	12	1	0.089	**
HOXA5	1	10	11.579	**
TMPRSS11B	1	10	11.579	**
SEL1L	9	0	0	**
FER1L6	10	25	3.001	**
OR52R1	14	2	0.153	**
HTT	2	12	6.986	**
NOVA1	3	14	5.463	**
CABIN1	0	7	Inf	**
CATSPERE	0	7	Inf	**
DNAAF1	0	7	Inf	**
IFNB1	0	7	Inf	**
INSYN1	0	7	Inf	**
STIP1	0	7	Inf	**
TTYH2	0	7	Inf	**
WDR44	0	7	Inf	**
WSCD1	0	7	Inf	**
RP1L1	32	51	1.996	**
COL12A1	19	36	2.316	**
TTN	97	116	1.668	**
OR4A15	10	24	2.867	**
MTCL1	5	16	3.754	**
TFAP2D	5	16	3.754	**
AKNA	1	9	10.375	**
OR10V1	1	9	10.375	**
SLC35F1	1	9	10.375	**
F2RL2	8	0	0	**
RP1	13	27	2.488	**
TRIP12	3	13	5.05	**
EPHA8	2	11	6.375	**
MAGEB1	2	11	6.375	**
NYNRIN	2	11	6.375	**
NF1	17	32	2.274	**
SEMA3A	6	17	3.326	**
UTRN	8	20	2.954	*
CARD8	0	6	Inf	*
CCNT2	0	6	Inf	*
GPCPD1	0	6	Inf	*
IRX3	0	6	Inf	*
KLHL20	0	6	Inf	*
NKAIN2	0	6	Inf	*
NR2E1	0	6	Inf	*
NTSR1	0	6	Inf	*
OPTN	0	6	Inf	*
OTOL1	0	6	Inf	*
PBX3	0	6	Inf	*
PLEKHB2	0	6	Inf	*
RADX	0	6	Inf	*
SEC31B	0	6	Inf	*
SLC10A3	0	6	Inf	*
TENM1	18	33	2.217	*
AKAP17A	10	1	0.108	*
CACNA2D4	5	15	3.503	*
SLITRK4	13	26	2.384	*
MRC1	22	37	2.041	*
RBM10	25	9	0.378	*
KALRN	11	24	2.596	*
TRRAP	11	24	2.596	*
PCDHGA6	7	18	3.022	*
FRMPD1	9	21	2.759	*
CTNNB1	14	3	0.23	*
PDCD11	14	3	0.23	*
NCOA6	4	14	4.083	*
PLD1	4	14	4.083	*
ROBO3	4	14	4.083	*
ABCB7	1	8	9.182	*
ANKK1	1	8	9.182	*
EZH2	1	8	9.182	*
HIPK3	1	8	9.182	*
MANEA	1	8	9.182	*
OR51F2	1	8	9.182	*
PIGG	1	8	9.182	*
SLC22A6	1	8	9.182	*
USP11	1	8	9.182	*
WDR62	1	8	9.182	*
RICTOR	3	12	4.642	*
SLC9A9	3	12	4.642	*
KRTAP13−1	7	0	0	*
PAQR9	7	0	0	*
PPP3R2	7	0	0	*
RBMX	7	0	0	*
EFCAB6	2	10	5.77	*
PRAMEF2	2	10	5.77	*
SLITRK5	6	16	3.117	*
LRRIQ1	15	28	2.229	*
OR4C46	8	19	2.793	*
LTBP1	16	29	2.166	*
TG	16	29	2.166	*
TP53	111	125	1.556	*
AHNAK2	23	37	1.944	*
KEAP1	57	32	0.568	*
IGHV1OR21−1	16	4	0.267	*
HCRTR2	5	14	3.255	*
OTOA	5	14	3.255	*
ROS1	5	14	3.255	*
ABCB11	7	17	2.841	*
ANO1	9	1	0.121	*
PPP3CA	9	1	0.121	*
SLC5A4	9	1	0.121	*
THSD4	9	1	0.121	*
DCLK1	13	3	0.249	*
BRAF	25	10	0.422	*
ANPEP	0	5	Inf	*
AP1M1	0	5	Inf	*
C16orf82	0	5	Inf	*
CACNG2	0	5	Inf	*
CCT6A	0	5	Inf	*
CD101	0	5	Inf	*
CSAG3	0	5	Inf	*
CYP2C18	0	5	Inf	*
CYSLTR1	0	5	Inf	*
DDX43	0	5	Inf	*
DOK1	0	5	Inf	*
EFTUD2	0	5	Inf	*
FAM13A	0	5	Inf	*
GABRR2	0	5	Inf	*
HAUS3	0	5	Inf	*
IL36A	0	5	Inf	*
LMBR1L	0	5	Inf	*
MAP9	0	5	Inf	*
MARCHF8	0	5	Inf	*
MED24	0	5	Inf	*
NAALADL1	0	5	Inf	*
NOC4L	0	5	Inf	*
NOM1	0	5	Inf	*
PPM1A	0	5	Inf	*
PPM1D	0	5	Inf	*
RECQL	0	5	Inf	*
RECQL4	0	5	Inf	*
RHOBTB1	0	5	Inf	*
RNASEH1	0	5	Inf	*
RNF138	0	5	Inf	*
RNF144B	0	5	Inf	*
SLC25A53	0	5	Inf	*
STKLD1	0	5	Inf	*
TAPT1	0	5	Inf	*
TBC1D10A	0	5	Inf	*
TOM1	0	5	Inf	*
TPI1	0	5	Inf	*
UBN1	0	5	Inf	*
UBR3	0	5	Inf	*
USP5	0	5	Inf	*
ZNF783	0	5	Inf	*
DOCK3	11	2	0.197	*
HRH2	11	2	0.197	*
PCDHGA10	11	2	0.197	*
LY75	4	13	3.775	*
OR51G2	4	13	3.775	*
THSD7B	15	27	2.139	*
EPHB1	10	21	2.474	*
HRNR	19	32	2.018	*
CHD8	6	15	2.909	*
PCDHGA8	6	15	2.909	*
SORCS1	23	36	1.882	*
ABCC12	3	11	4.236	*
GALNTL6	3	11	4.236	*
NR5A2	3	11	4.236	*
OR14I1	3	11	4.236	*
SLC1A3	3	11	4.236	*
SLC8A3	17	5	0.314	*
CPS1	20	33	1.979	*
BUB1	2	9	5.17	*
EVI5	2	9	5.17	*
KDM4C	2	9	5.17	*
KRT37	2	9	5.17	*
OR8D4	2	9	5.17	*
PLEKHH2	2	9	5.17	*
UBE3A	2	9	5.17	*
WDPCP	2	9	5.17	*
TEX15	17	29	2.031	*
ASIC5	1	7	7.999	*
CEP85L	1	7	7.999	*
CFHR2	1	7	7.999	*
COLEC11	1	7	7.999	*
CPA6	1	7	7.999	*
FAM78B	1	7	7.999	*
GLYAT	1	7	7.999	*
KMO	1	7	7.999	*
KMT2E	1	7	7.999	*
KRT28	1	7	7.999	*
OR2V2	1	7	7.999	*
PIP4K2C	1	7	7.999	*
POLR1B	1	7	7.999	*
SETDB1	1	7	7.999	*
SLAMF1	1	7	7.999	*
SLC17A1	1	7	7.999	*
TTLL4	1	7	7.999	*
NLGN1	9	19	2.473	*
SELP	9	19	2.473	*
ATAD2B	6	0	0	*
DCAF13	6	0	0	*
KRT16	6	0	0	*
PDLIM5	6	0	0	*
PPP4R4	6	0	0	*
PRDM1	6	0	0	*
RAB11FIP2	6	0	0	*
ROCK2	6	0	0	*
RRP1B	6	0	0	*
SH3RF1	6	0	0	*
TICRR	6	0	0	*
TONSL	6	0	0	*
ZHX2	6	0	0	*
ZNRF4	6	0	0	*
DPY19L2	14	4	0.308	*
NLRC5	14	4	0.308	*
ADAMTS3	15	4	0.286	*
BMPER	15	4	0.286	*
ABCC9	7	16	2.662	*
AMPH	7	16	2.662	*
COL15A1	7	16	2.662	*
ACSM5	18	6	0.356	*
CROCC	12	3	0.27	*
IMPG2	12	3	0.27	*
MTR	12	3	0.27	*
CDH17	4	12	3.469	*
FN1	4	12	3.469	*
OR10R2	4	12	3.469	*
PALLD	4	12	3.469	*
STYXL2	25	11	0.467	*
FKBP6	10	2	0.217	*
GPC5	10	2	0.217	*
LARP4B	10	2	0.217	*
MBL2	10	2	0.217	*
PRLR	10	2	0.217	*
AMER2	8	1	0.136	*
CEACAM5	8	1	0.136	*
CLIP1	8	1	0.136	*
FASLG	8	1	0.136	*
HSF5	8	1	0.136	*
PACRG	8	1	0.136	*
SERGEF	8	1	0.136	*
SLC9B1	8	1	0.136	*
SPATA6	8	1	0.136	*
CTCFL	6	14	2.703	*
LRRK2	6	14	2.703	*
OR5J2	6	14	2.703	*
YLPM1	6	14	2.703	*
CHL1	14	24	2.016	*
MYO18B	14	24	2.016	*
WDFY3	14	24	2.016	*
BPI	3	10	3.834	*
CTAGE1	3	10	3.834	*
FPGT−TNNI3K	3	10	3.834	*
GPR37	3	10	3.834	*
MAGEA12	3	10	3.834	*
PHF20L1	3	10	3.834	*
TMPRSS11F	3	10	3.834	*
TRAPPC9	3	10	3.834	*
ZKSCAN2	3	10	3.834	*
RYR3	32	44	1.658	*
FSCB	9	18	2.333	*
ITIH6	9	18	2.333	*
REG3A	9	18	2.333	*
PTPRZ1	15	25	1.962	*

Odds ratio with 95% CI
(1 = no effect, < 1 High Epithelial_cells has more mutants)
