# Supplementary material for: Integrative multi-omics analysis for identifying novel therapeutic targets and predicting immunotherapy efficacy in lung adenocarcinoma
Source: Cancer Drug Resist. 2025 Jan 14;8:3. doi: 10.20517/cdr.2024.91 (PMC11810459; doi:10.20517/cdr.2024.91)
Supplement: Supplementary file 1 [file cdr-8-3-SupplementaryFiles.zip › cdr7091-SupplementaryFiles/Supplementary Figures/Supplementary Figure 4.pdf]

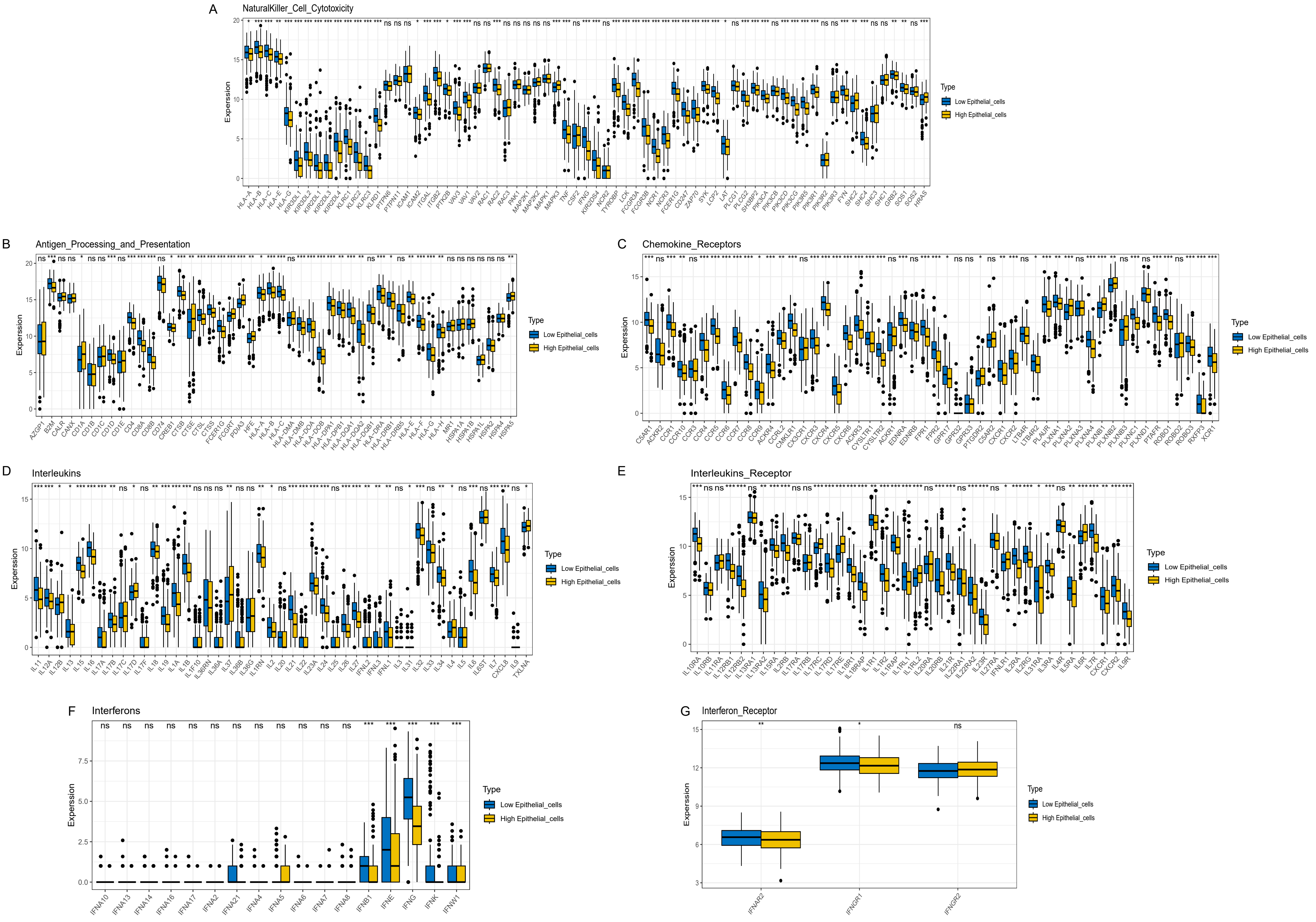

A
NaturalKiller_Cell_Cytotoxicity
Expersssion
HLA-A HLA-B HLA-C HLA-E HLA-G KIR3DL1 KIR3DL2 KIR2DL1 KIR2DL3 KIR2DL4 KLRC1 KLRC2 KLRC3 KLRD1 PTPN6 PTPN11 ICAM1 ICAM2 ITGAL ITGB2 PTK2B VAV3 VAV1 VAV2 RAC1 RAC2 RAC3 PAK1 MAP2K1 MAP2K2 MAPK1 MAPK3 TNF CSF2 IFNG KIR2DS4 NCR2 TYROBP LCK FCGR3A FCGR3B NCR1 NCR3 FCER1G CD247 ZAP70 SYK LCP2 LAT PLCG1 PLCG2 SH3BP2 PIK3CA PIK3CB PIK3CD PIK3CG PIK3R5 PIK3R1 PIK3R2 PIK3R3 FYN SHC2 SHC4 SHC3 SHC1 GRB2 SOS1 SOS2 HRAS
Type
Low Epithelial_cells
High Epithelial_cells
B
Antigen_Processing_and_Presentation
Expersssion
AZGP1 B2M CALR CANX CD1A CD1B CD1C CD1D CD1E CD4 CD8A CD8B CD74 CREB1 CTSB CTSE CTSL CTSS FCER1G FCGRT PDIA3 HFE HLA-A HLA-B HLA-C HLA-DMA HLA-DMB HLA-DOA HLA-DOB HLA-DPA1 HLA-DPB1 HLA-DQA1 HLA-DQA2 HLA-DQB1 HLA-DRA HLA-DRB1 HLA-DRB5 HLA-E HLA-F HLA-G HLA-H MR1 HSPA1A HSPA1B HSPA1L HSPA2 HSPA4 HSPA5
Type
Low Epithelial_cells
High Epithelial_cells
C
Chemokine_Receptors
Expersssion
C5AR1 ACKR2 CCR1 CCR10 CCR3 CCR4 CCR5 CCR6 CCR7 CCR8 CCR9 ACKR4 CCRL2 CMKLR1 CX3CR1 CXCR3 CXCR4 CXCR5 CXCR6 ACKR3 CYSLTR1 CYSLTR2 ACKR1 EDNRA EDNRB FPR1 FPR2 GPR17 GPR32 GPR33 PTGDR2 C5AR2 CXCR1 CXCR2 LTB4R LTB4R2 PLAUR PLXNA1 PLXNA2 PLXNA3 PLXNA4 PLXNB1 PLXNB2 PLXNB3 PLXNC1 PLXND1 PTAFR ROBO1 ROBO2 ROBO3 RXFP3 XCR1
Type
Low Epithelial_cells
High Epithelial_cells
D
Interleukins
Expersssion
IL11 IL12A IL12B IL13 IL15 IL16 IL17A IL17B IL17C IL17D IL17F IL18 IL19 IL1A IL1B IL1F10 IL36RN IL36A IL37 IL36B IL36G IL1RN IL2 IL20 IL21 IL22 IL23A IL24 IL25 IL26 IL27 IFNL2 IFNL3 IFNL1 IL3 IL31 IL32 IL33 IL34 IL4 IL5 IL6 IL6ST IL7 CXCL8 IL9 TXLNA
Type
Low Epithelial_cells
High Epithelial_cells
E
Interleukins_Receptor
Expersssion
IL10RA IL10RB IL11RA IL12RB1 IL12RB2 IL13RA1 IL13RA2 IL15RA IL2RB IL17RA IL17RB IL17RC IL17RD IL17RE IL18R1 IL18RAP IL1R1 IL1R2 IL1RAP IL1RL1 IL1RL2 IL20RA IL20RB IL21R IL22RA1 IL22RA2 IL23R IL27RA IFNLR1 IL2RA IL2RG IL31RA IL3RA IL4R IL5RA IL6R IL7R CXCR1 CXCR2 IL9R
Type
Low Epithelial_cells
High Epithelial_cells
F
Interferons
ns ns ns ns ns ns ns ns ns ns ns ns *** *** *** *** ***
Expersssion
IFNA10 IFNA13 IFNA14 IFNA16 IFNA17 IFNA2 IFNA21 IFNA4 IFNA5 IFNA6 IFNA7 IFNA8 IFNB1 IFNE IFNG IFNK IFNW1
Type
Low Epithelial_cells
High Epithelial_cells
G
Interferon_Receptor
** * ns
Expersssion
IFNAR2 IFNGR1 IFNGR2
Type
Low Epithelial_cells
High Epithelial_cells
