# Supplementary material for: Integrative multi-omics analysis for identifying novel therapeutic targets and predicting immunotherapy efficacy in lung adenocarcinoma
Source: Cancer Drug Resist. 2025 Jan 14;8:3. doi: 10.20517/cdr.2024.91 (PMC11810459; doi:10.20517/cdr.2024.91)
Supplement: Supplementary file 1 [file cdr-8-3-SupplementaryFiles.zip › cdr7091-SupplementaryFiles/Supplementary Materials.pdf]

**Supplementary Materials**

# Integrative multi-omics analysis for identifying novel therapeutic targets and predicting immunotherapy efficacy in lung adenocarcinoma

**Kun Mei[1,2,#], Zilu Chen[1,#], Foxing Tan[1], YuHeng Zhou[1], Haolin Du[1], Min Wang[2], Renjun Gu[3,4], Yan Huang[5]**

[1]Nanjing University of Chinese Medicine, Nanjing 210023, Jiangsu, China.
[2]Department of Cardiothoracic Surgery, The Third Affiliated Hospital of Soochow University, Changzhou 213003, Jiangsu, China.
[3]School of Chinese Medicine and School of Integrated Chinese and Western Medicine, Nanjing University of Chinese Medicine, Nanjing 210023, Jiangsu, China.
[4]Jinling Hospital, Affiliated Hospital of Medical School, Nanjing University, Nanjing 210046, Jiangsu, China.
[5]Department of Ultrasound, Nanjing Hospital of Chinese Medicine Affiliated with Nanjing University of Chinese Medicine, Nanjing 210022, Jiangsu, China.
[#]Authors contributed equally.

**Correspondence to:** Prof. Yan Huang, Department of Ultrasound, Nanjing Hospital of Chinese Medicine Affiliated with Nanjing University of Chinese Medicine, No. 157 Daming Road, Nanjing 210022, Jiangsu, China. E-mail: jacob6666@163.com. Prof. Renjun Gu, School of Chinese Medicine and School of Integrated Chinese and Western Medicine, Nanjing University of Chinese Medicine, No. 138 Xianlin Avenue, Nanjing 210023, Jiangsu, China. E-mail: renjungu@hotmail.com. Prof. Min Wang, Department of Cardiothoracic Surgery, The Third Affiliated Hospital of Soochow University, No. 185 Guqian Street, Changzhou 213003, Jiangsu, China. E-mail: wangmin80808@163.com

**Supplementary Materials** detailed materials and methods

## Materials and Method

### Data retrieval and preprocessing

We first downloaded multi-omics data of LUAD from the TCGA database (https://tcga-data.nci.nih.gov/), including complete transcriptome expression data, DNA methylation, somatic mutations, and matching clinical data. Transcriptomic profiles of mRNA and lncRNA were obtained through the TCGAbiolinks package. The IDs of mature miRNAs in TCGA were recorded through the miRBaseVersions.db package. Somatic mutations were also obtained through TCGAbiolinks and processed using the maftools package. DNA methylation profiles and clinical information were downloaded from UCSC Xena (https://xenabrowser.net/). After downloading, the data was processed using the robust limma package for correction, log2 transformation, and data normalization. Subsequently, we downloaded the LUAD single-cell sequencing dataset (GSE189357) and LUAD transcriptome datasets (GSE31210, GSE50081) from the GEO database and used the SVA package for batch effect removal and merging. Additionally, we downloaded three immunotherapy datasets (GSE78220, GSE91061, GSE135222) for subsequent immune analysis and obtained clinical trial data on immunotherapy efficacy from http://research-pub.gene.com/IMvigor210CoreBiologies.
The GWAS data (ieu-a-984) on LUAD was downloaded from the IEU OPEN GWAS database (https://gwas.mrcieu.ac.uk/), which included data from 65,864 Europeans, consisting of 11,245 lung adenocarcinoma patients and 54,619 controls.

### Single-cell sequencing analysis

We obtained the raw single-cell sequencing data (GSE189357), which included samples from nine LUAD patients. Before analysis, we excluded low-quality cells through quality control, eliminating cells with fewer than 200 expressed genes or mitochondrial gene proportions exceeding 20%. Subsequently, we used Harmony for batch correction and dimensionality reduction, selecting PC=30 for further analysis and a resolution of 0.6 for cell clustering. After clustering, cell populations were annotated using the singleR package. Finally, we used the CellChat package to predict intercellular communication patterns among all identified cell types.

### Bayesian deconvolution of cell types and gene expression

Bayesian deconvolution uses a reference scRNA-seq to infer two statistics for each bulk RNA-seq sample: (1) the proportion of reads from each cell type, assuming it is proportional to the proportion of that cell type; (2) the gene expression levels of each cell type. The most challenging aspect of cellular deconvolution is accounting for various sources of uncertainty, including technical and biological batch variations and gene expression differences between bulk and reference scRNA-seq. To address these uncertainties, we adopted a Bayesian approach, modeling prior distributions with scRNA-seq and using observed data to infer the joint posterior distribution of cell type proportions and gene expression in each bulk sample. Thus, uncertainties in each estimate could be drawn from the joint posterior.

We first identified disease-associated cell subsets at the single-cell level using the scPagwas package, which performs polygenic linear regression of pathway activity scores from scRNA-seq data with GWAS genetic signals to identify trait-related genes for inferring trait-related cell subsets. Through extensive simulations and real data evaluations, many well-known cell type-disease associations were replicated, and disease-related cell subsets were newly discovered. By scoring LUAD-related cell subsets and calculating a trait-relevant score (TRS) for each subset, we obtained trait-related genes. Using the processed single-cell count matrix, single-cell annotation files, and TCGA transcriptome data, we performed deconvolution analysis using the BayesPrism package, obtaining scores for each cell subset in each sample for subsequent analysis.

**Identification of functional differences in convoluted cell subsets**

We first normalized each sample's subsets, identifying tumor versus control differences between transcription profiles of each subset. Subsequently, we performed differential analysis on convoluted cells using the DESeq2 package, identifying group differences and visualizing them through heatmaps and volcano plots. Using the ImmPort database (https://immport.org/shared/), we identified differences in immune functions between related cell subsets. Simultaneously, we conducted immunotherapy differences analysis on cell subsets using mimiconda software and performed drug prediction on designated convoluted cells using the oncoPredict package. To further verify the differences in convoluted cell subsets, we performed somatic mutation analysis on cell subsets using the maftools package and calculated the percentage of

the genome with copy number alterations using copy number segment data. Finally, we identified key module genes in convoluted cells using the WGCNA package. To determine the optimal soft-thresholding power, we employed the scale-free topology criterion. Subsequently, transformations of the weighted adjacency matrix and the topological overlap matrix were generated. Hierarchical clustering and tree analysis were conducted to filter modules containing more than 50 genes.

**Multi-omics integration analysis**

In this study, we extracted TCGA-LUAD multi-omics data, including mRNA, lncRNA, miRNA, and methylation. We used the MOVICS package to screen the top 1500 genes with the highest variation and combined clinical data to identify prognostic genes ($p < 0.05$). Subsequently, we used the maftools package to screen the top 5000 genes with the highest mutation rates and finally identified the top 5% most common mutation genes through the method parameter. These data results were incorporated into our research for further analysis.

To further determine our optimal clustering number, we used 10 clustering algorithms (CIMLR, ConsensusClustering, SNF, iClusterBayes, PINSPlus, moCluster, NEMO, IntNMF, COCA, and LRA), obtaining clustering results for each algorithm, and based on consensus clustering, we finally decided to divide them into two subtypes.

**Molecular landscape of consensus clustering**

To calculate LUAD subtype-related features and different treatment-related features, we used gene set variation analysis (GSVA) for identification. Subsequently, we compared the characteristics of targeted therapy and radiotherapy and the distribution of immune checkpoints between LUAD subtypes and used the ESTIMATE package to evaluate the immune/stromal scores of tumor tissues. Additionally, we calculated DNA methylation scores based on the status of tumor-infiltrating lymphocytes (MeTIL). We then performed differential analysis between the two subtypes, selecting the top 100 upregulated genes in each subtype as features, and visualized the heatmap between subtypes using the ComplexHeatmap package. These genes served as classifiers to subtype the validation set and plotted Kaplan-Meier survival curves. We also evaluated the enrichment of 24 tumor immune microenvironment cells using

GSVA. Finally, we verified the consistency of consensus clustering using external datasets.

**Machine learning construction of prognostic features and clinical application of consensus clustering**

We first performed univariate prognostic analysis on the top 100 upregulated genes, then intersected them with the hub module genes identified by WGCNA, screening out 30 key hub prognostic genes. Using the TCGA dataset as the training set and the META dataset obtained by batch-effect removal merging of GSE31210 and GSE50081 using the SVA package as the validation set, we constructed the MOMLS with high accuracy and extensive generalizability using 10 machine learning algorithms, including CoxBoost, stepwise Cox, Lasso, Ridge, elastic net (Enet), survival support vector machines (survival-SVMs), generalized boosted regression models (GBMs), supervised principal components (SuperPC), partial least Cox (plsRcox), and RSF. Regardless of the training set or validation set, we used the C-index to predict the best performance of MOMLS, and only models with the highest C-index in both sets were considered optimal.

Based on the model, we scored each sample in the training and validation sets and divided them into high MOMLS and low MOMLS groups based on the scores. We evaluated the prognostic significance of MOMLS using Kaplan-Meier survival curves. To enhance the clinical utility of MOMLS, we constructed a nomogram using factors obtained from multivariate Cox regression. We plotted the time-dependent C-index curve and calibration curve to describe accuracy and used decision curves to calculate patients' clinical benefits.

**Immunological characterization and comprehensive analysis of immunotherapy response based on MOMLS**

We analyzed TME cell types, immunotherapy response, and immunosuppressive and immune rejection-related features in high MOMLS and low MOMLS groups using the IOBR package. Using a unified method, we calculated the enrichment scores for each sample, comprehensively analyzing the immunological differences between high MOMLS and low MOMLS groups. We compared differences in immune cell distribution between the two groups. For immunotherapy response, we first evaluated

the delayed response survival of patients to immunotherapy and estimated the immunotherapy response by combining the TIP algorithm, subclass mapping, and TIDE algorithm.

**Screening potential therapeutic drugs for MOMLS patients**

We analyzed the status of carcinogenic pathways in high MOMLS and low MOMLS groups using the GSEA algorithm. Human cancer cell line (CCL) expression data were obtained from the Cancer Cell Line Encyclopedia (CCLE) of the Broad Institute. CTRP v.2.0 (https://portals.broadinstitute.org/ctrp) and PRISM Repurposing datasets (19Q4; https://depmap.org/portal/prism/) were used to obtain drug sensitivity data for CCLs. The area under the dose-response curve (AUC) value was used as a measure of drug sensitivity.

**Colocalization analysis**

To prevent different but related causal variations between exposure and outcome, for results exceeding the MR threshold (FDR $<0.05$), we performed colocalization analysis using the COLOC package. Colocalization assesses the probability of a shared causal variant (PP.H4) or distinct causal variants (PP.H3) between the LUAD-GWAS and cis-pQTL instruments for the protein of interest. We performed conditional analysis on the pQTL data to identify conditionally distinct pQTL signals and performed colocalization using marginal (unadjusted) pQTL results as well as results conditional on each of the instruments used in the MR. Statistically significant MR hits with a posterior probability of a shared causal variant (PP.H4) $>0.5$ for at least one instrumental variant were then investigated further. The tissue used was lung tissue from GTEx V8.

**Statistical analysis**

All statistical and bioinformatics analyses were performed using R software. Continuous data were compared using t-tests or Mann-Whitney tests as appropriate. Analysis of different clinical outcomes was based on Kaplan-Meier plots and Cox regression analysis. Multi-omics integration analysis was performed using the MOVICS package. Statistical significance was determined at a p-value threshold of less than 0.05.
