# Supplementary figures and images for: Integrative multi-omics analysis for identifying novel therapeutic targets and predicting immunotherapy efficacy in lung adenocarcinoma
Source: Cancer Drug Resist. 2025 Jan 14;8:3. doi: 10.20517/cdr.2024.91 (PMC11810459; doi:10.20517/cdr.2024.91)

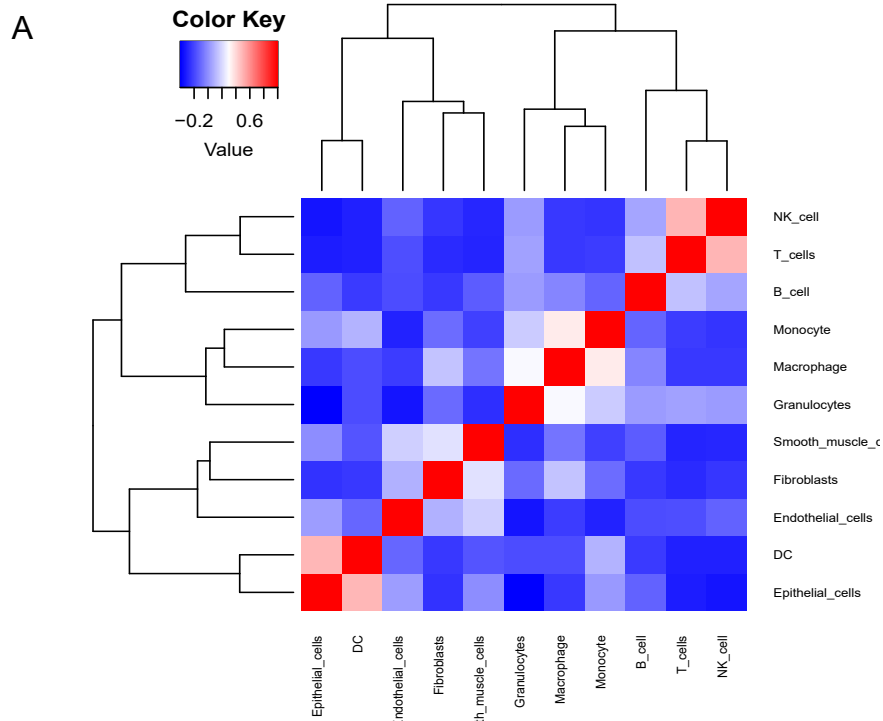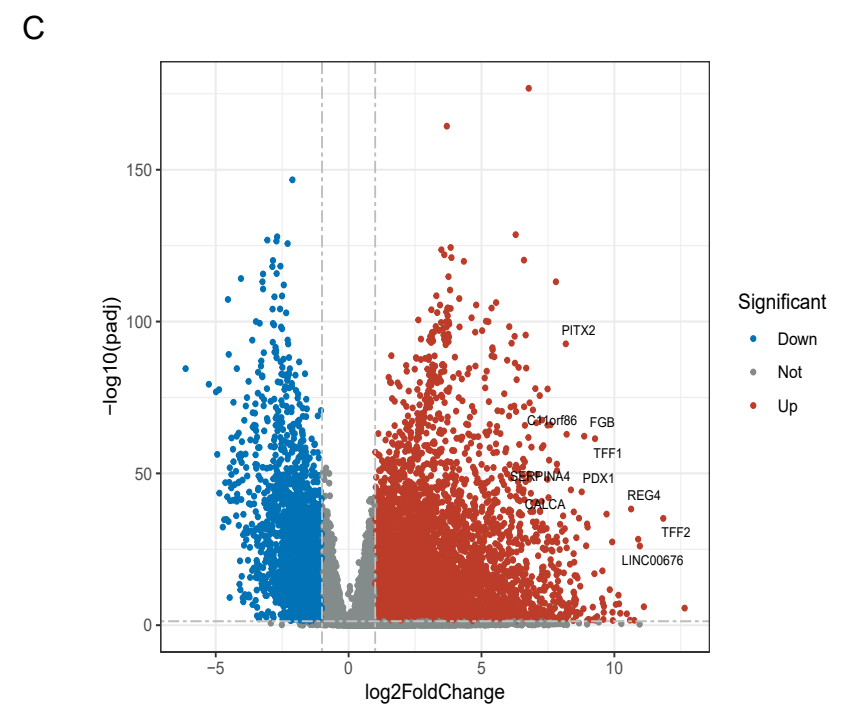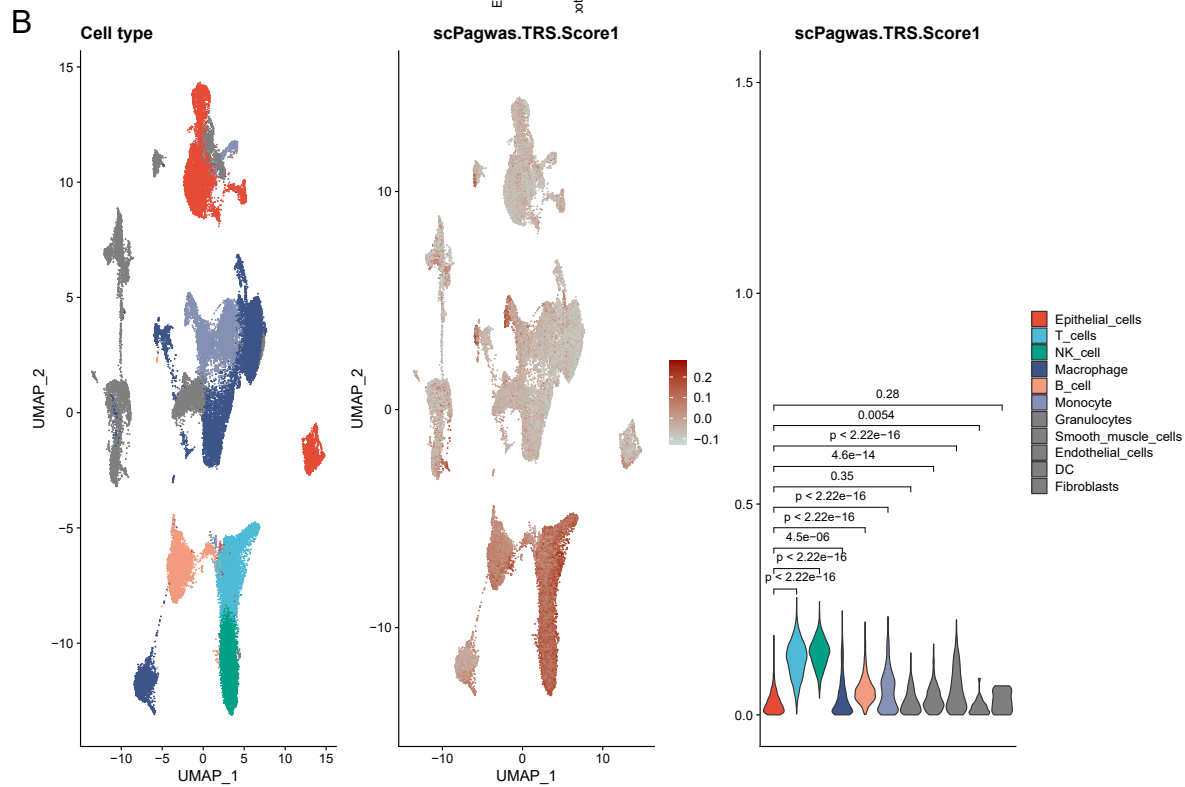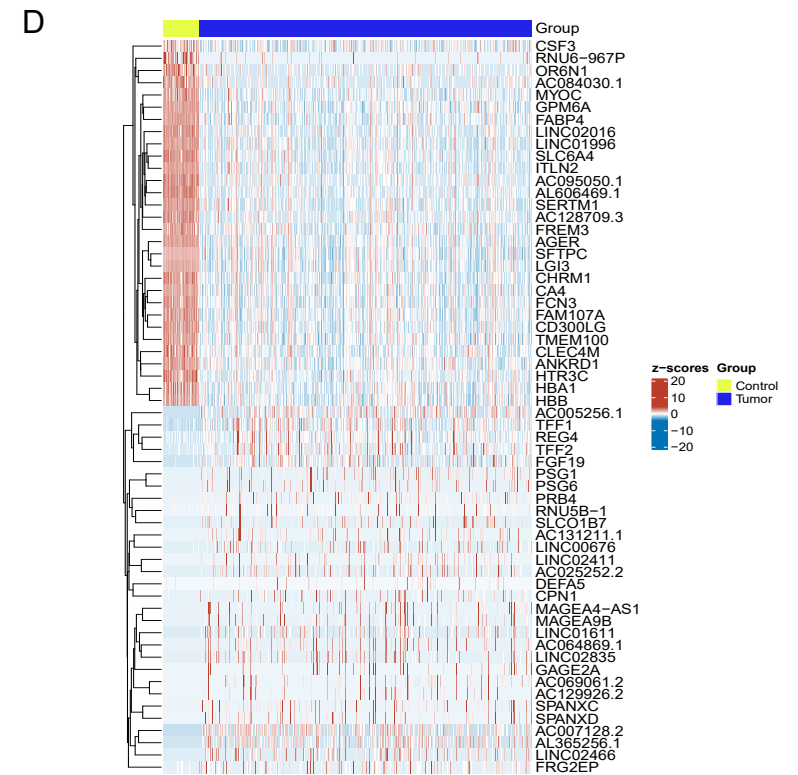

Supplement: Supplementary file 1 [file cdr-8-3-SupplementaryFiles.zip › cdr7091-SupplementaryFiles/Supplementary Figures/Supplementary Figure 1.pdf]

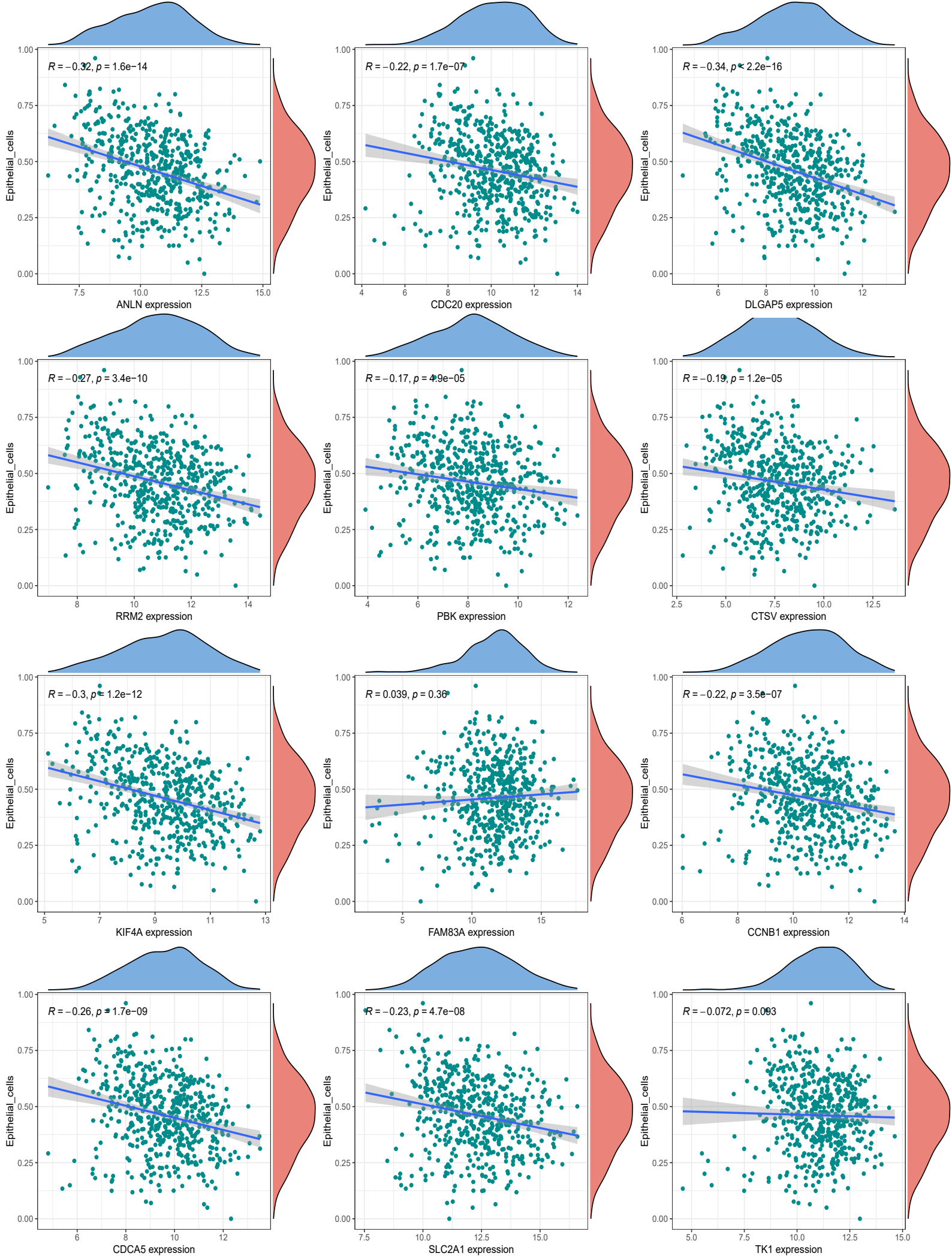

Supplement: Supplementary file 1 [file cdr-8-3-SupplementaryFiles.zip › cdr7091-SupplementaryFiles/Supplementary Figures/Supplementary Figure 10.pdf]

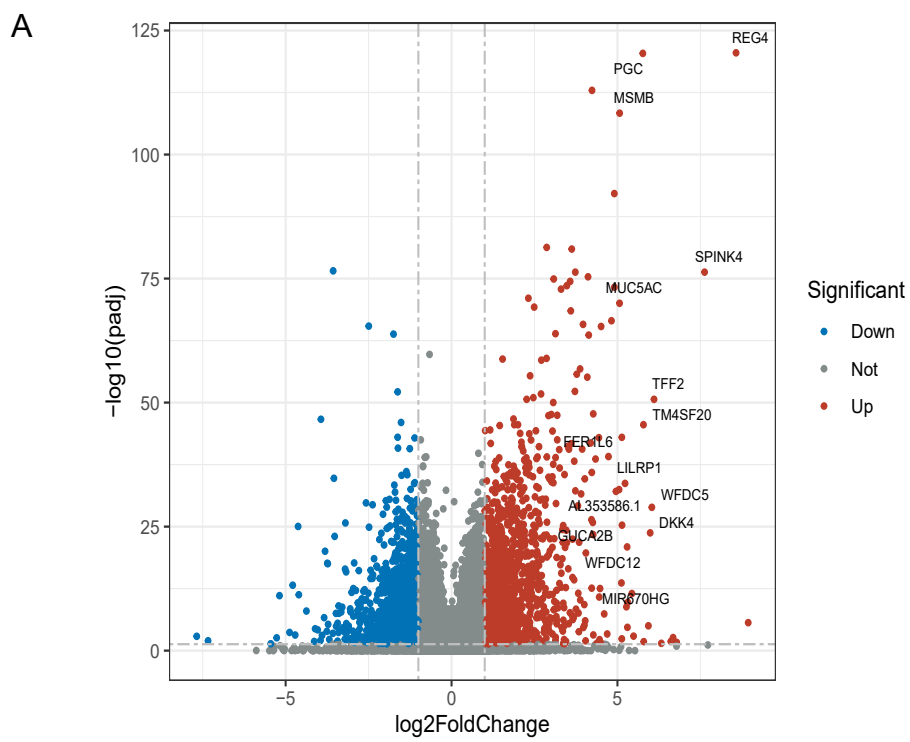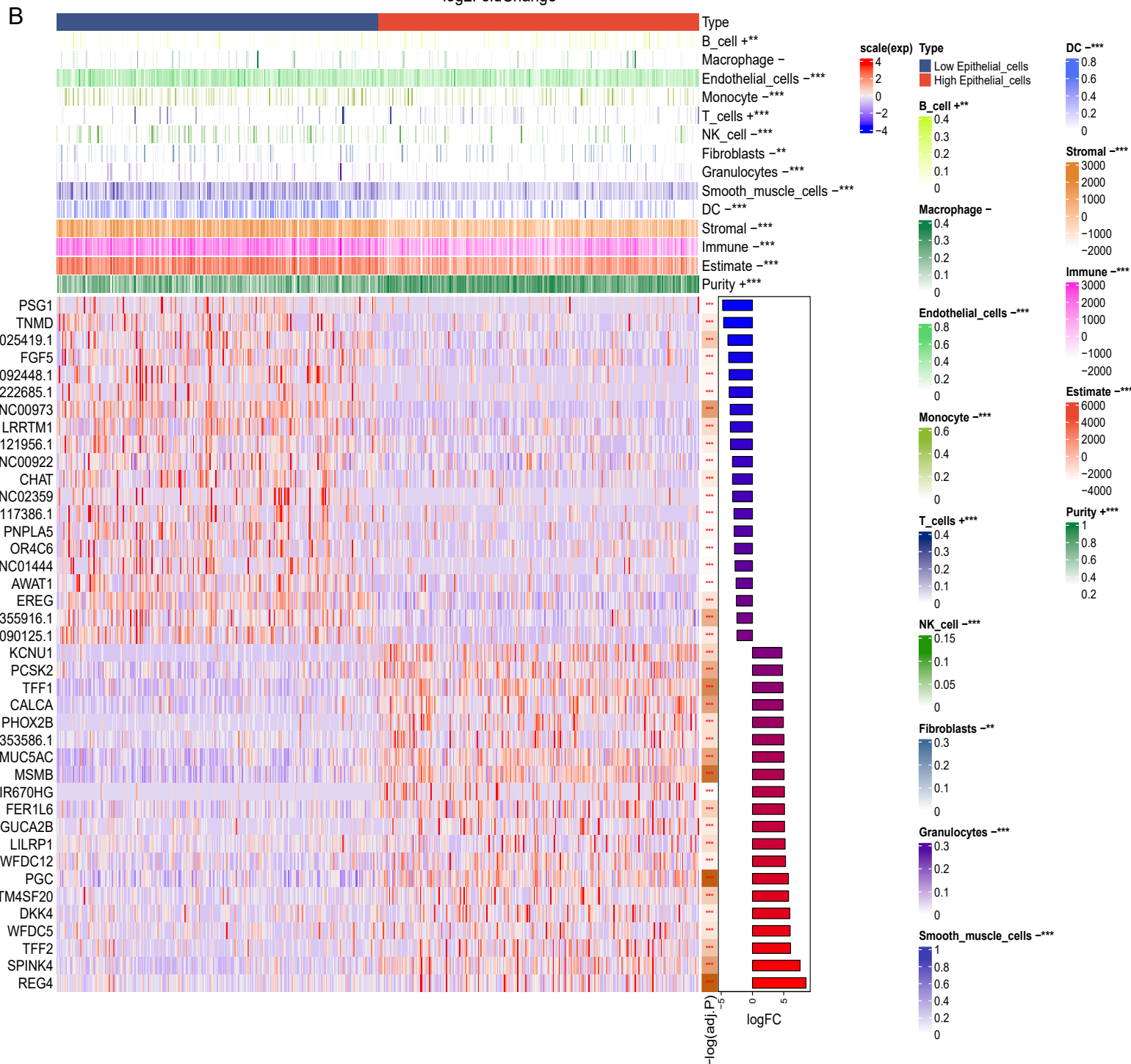

Supplement: Supplementary file 1 [file cdr-8-3-SupplementaryFiles.zip › cdr7091-SupplementaryFiles/Supplementary Figures/Supplementary Figure 2.pdf]

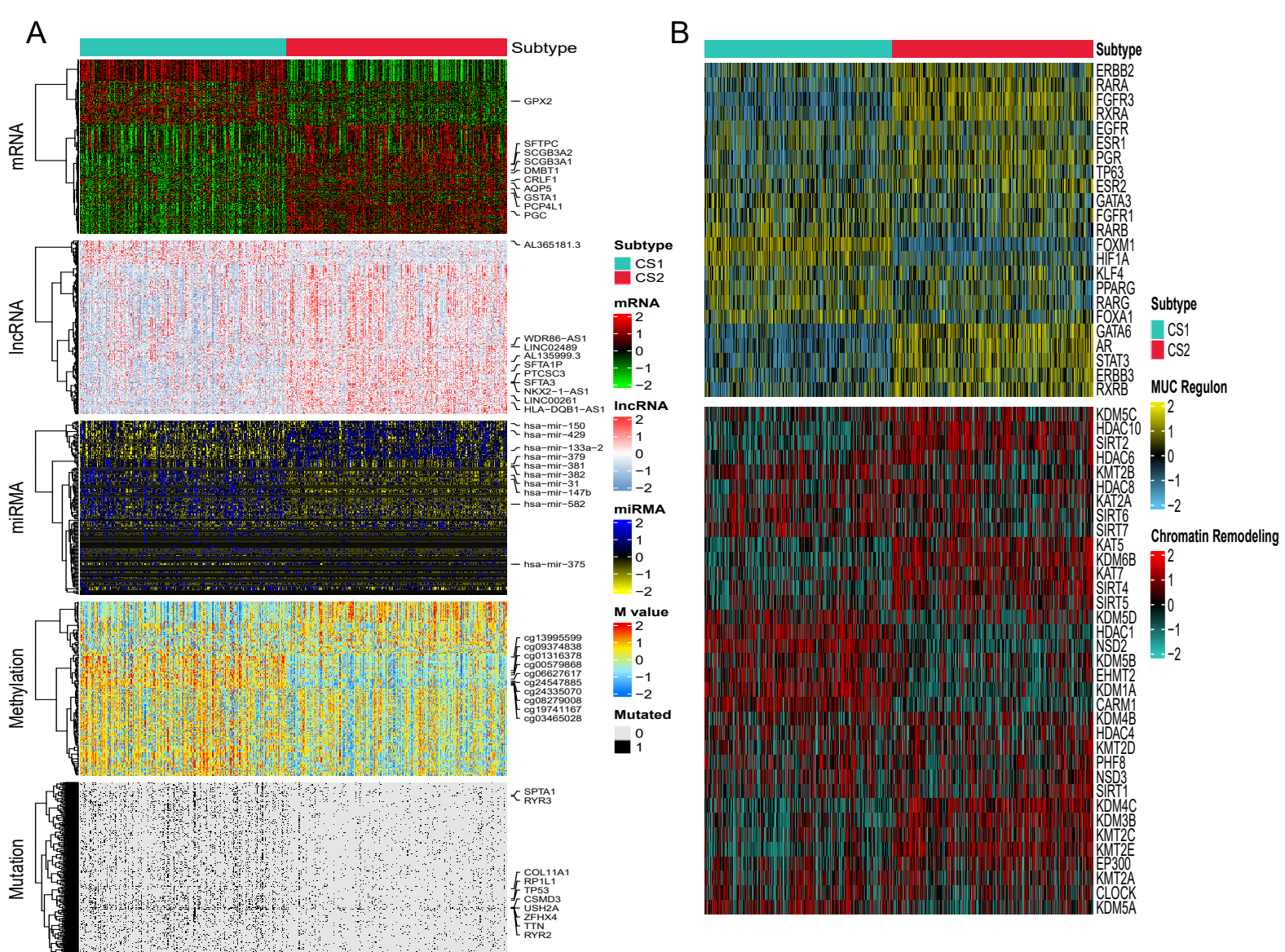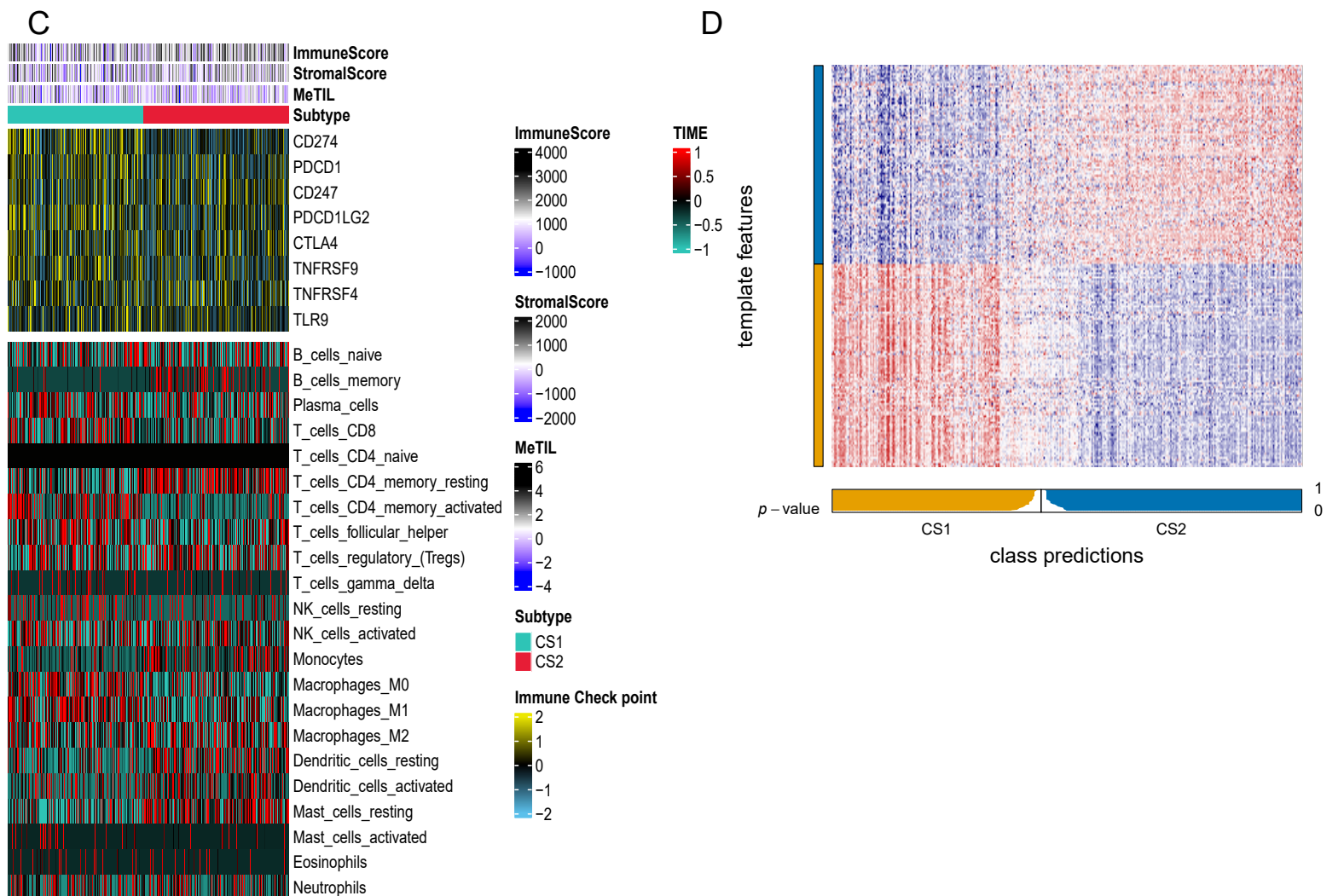

Supplement: Supplementary file 1 [file cdr-8-3-SupplementaryFiles.zip › cdr7091-SupplementaryFiles/Supplementary Figures/Supplementary Figure 5.pdf]

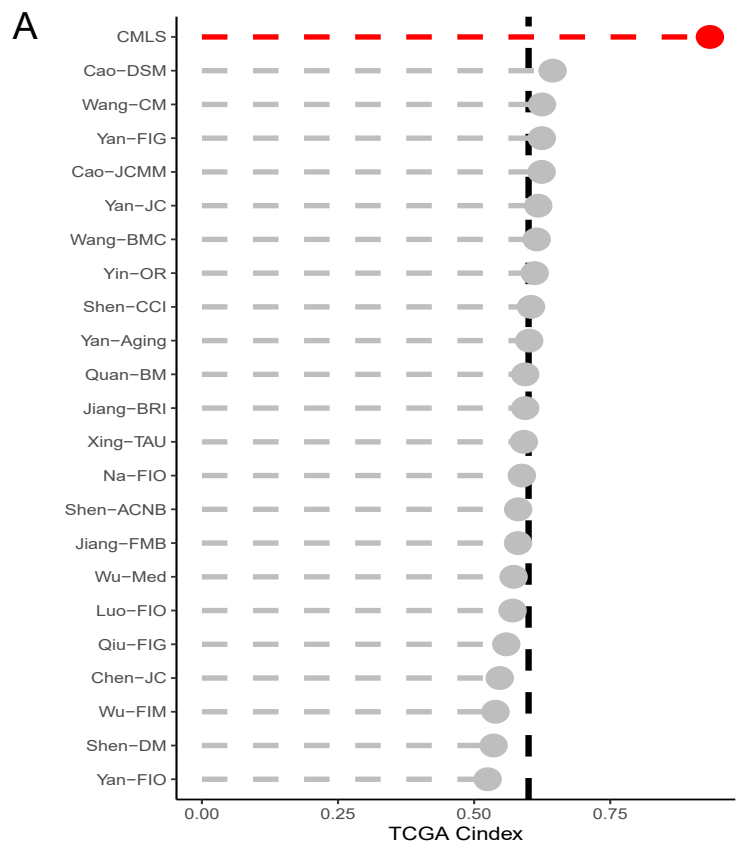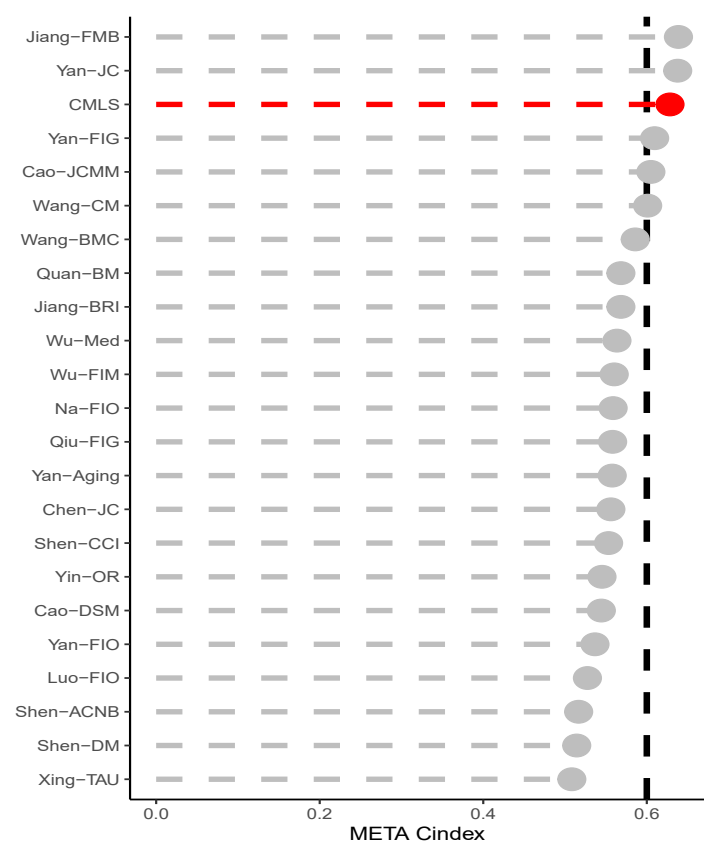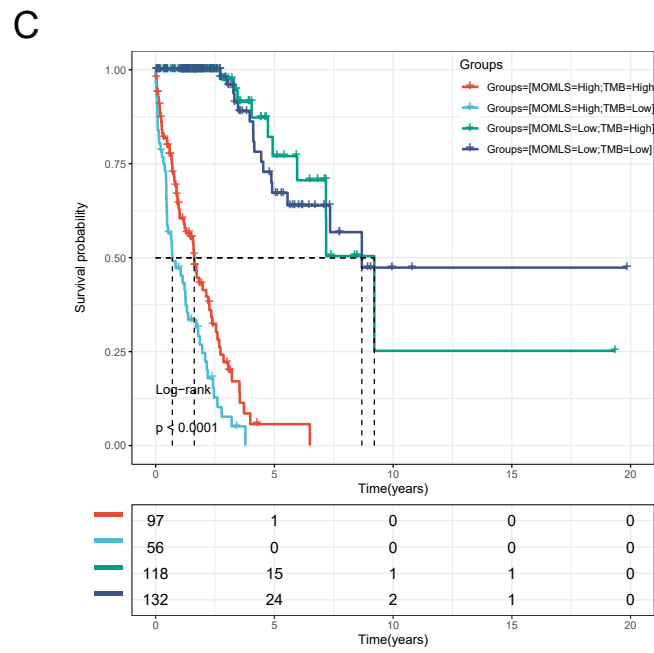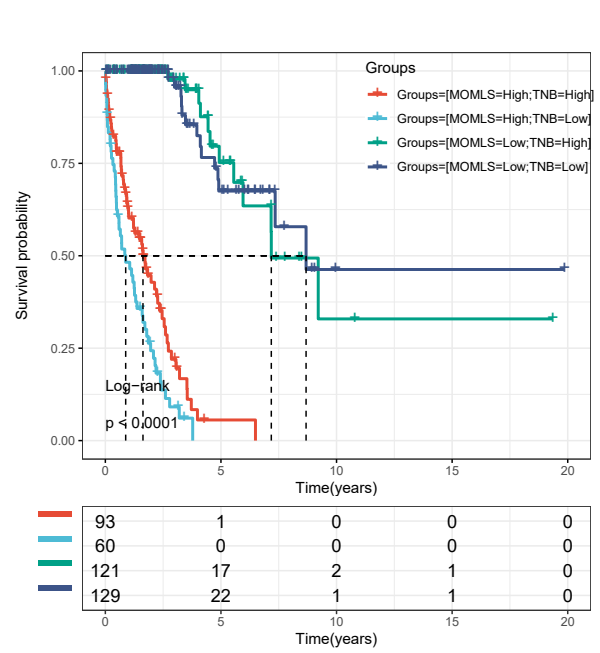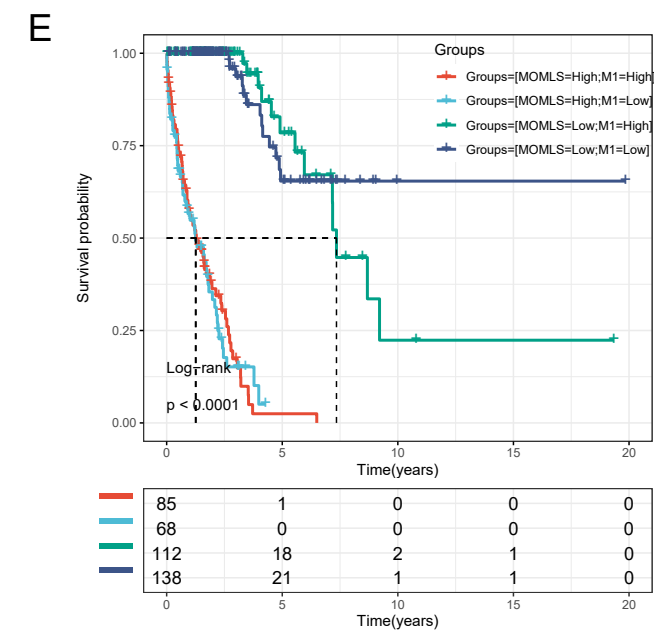

Supplement: Supplementary file 1 [file cdr-8-3-SupplementaryFiles.zip › cdr7091-SupplementaryFiles/Supplementary Figures/Supplementary Figure 6.pdf]

A

## tme\_cell\_types

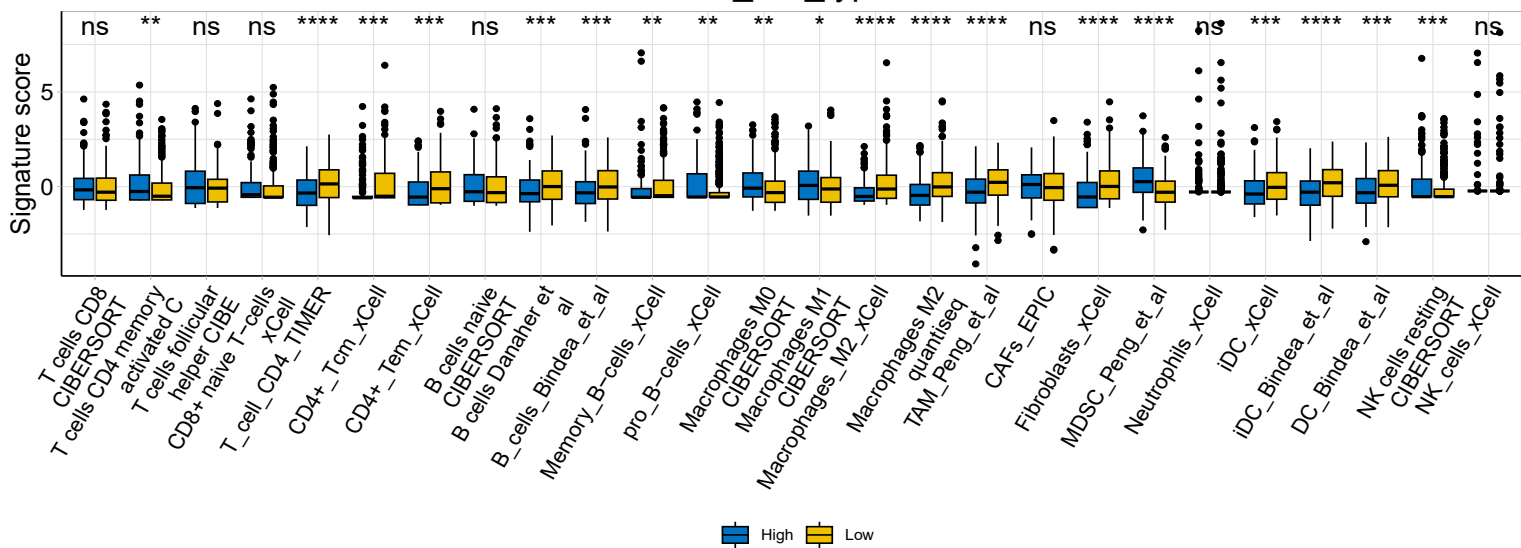

B

## immu\_exclusion

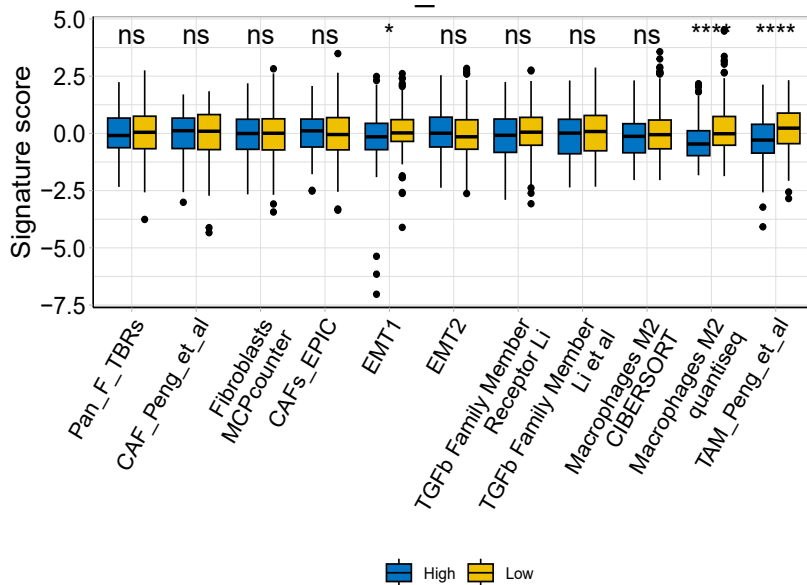

C

## immu\_suppression

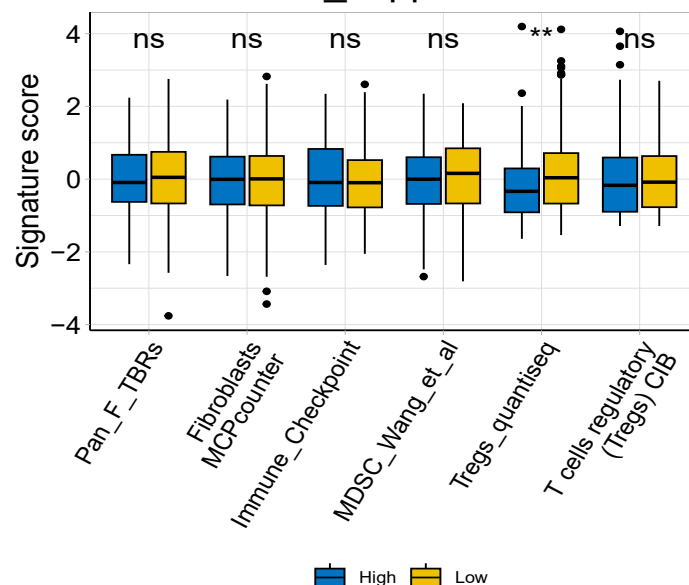

D

## io\_biomarkers

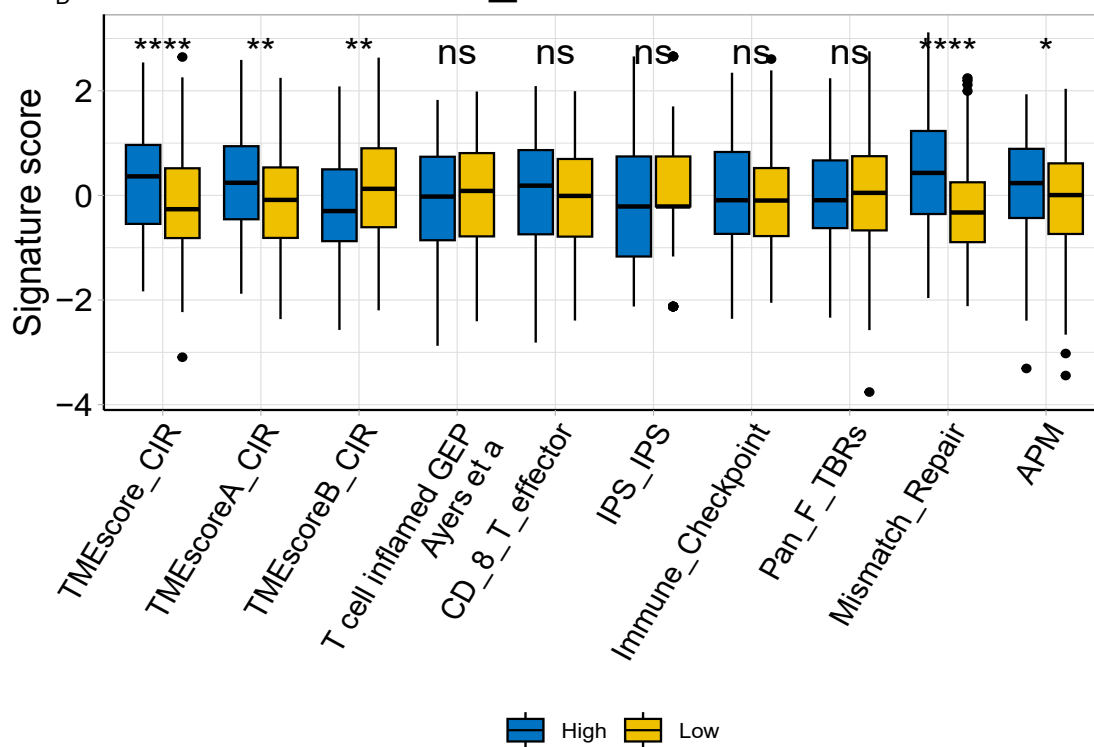

Supplement: Supplementary file 1 [file cdr-8-3-SupplementaryFiles.zip › cdr7091-SupplementaryFiles/Supplementary Figures/Supplementary Figure 7.pdf]

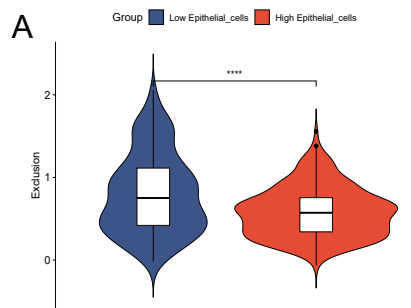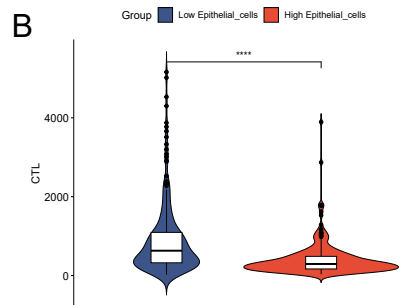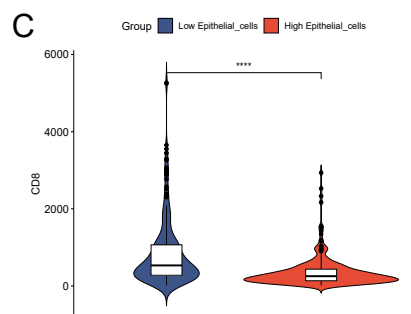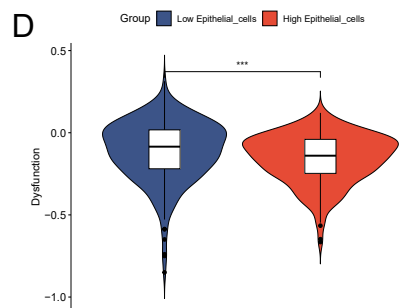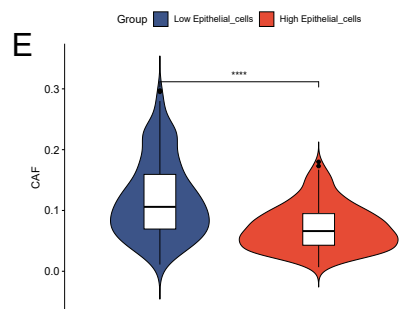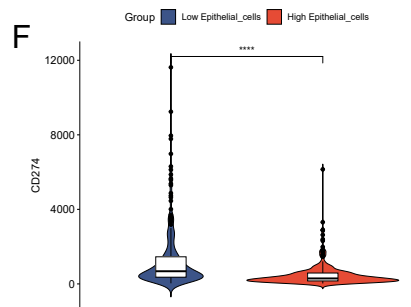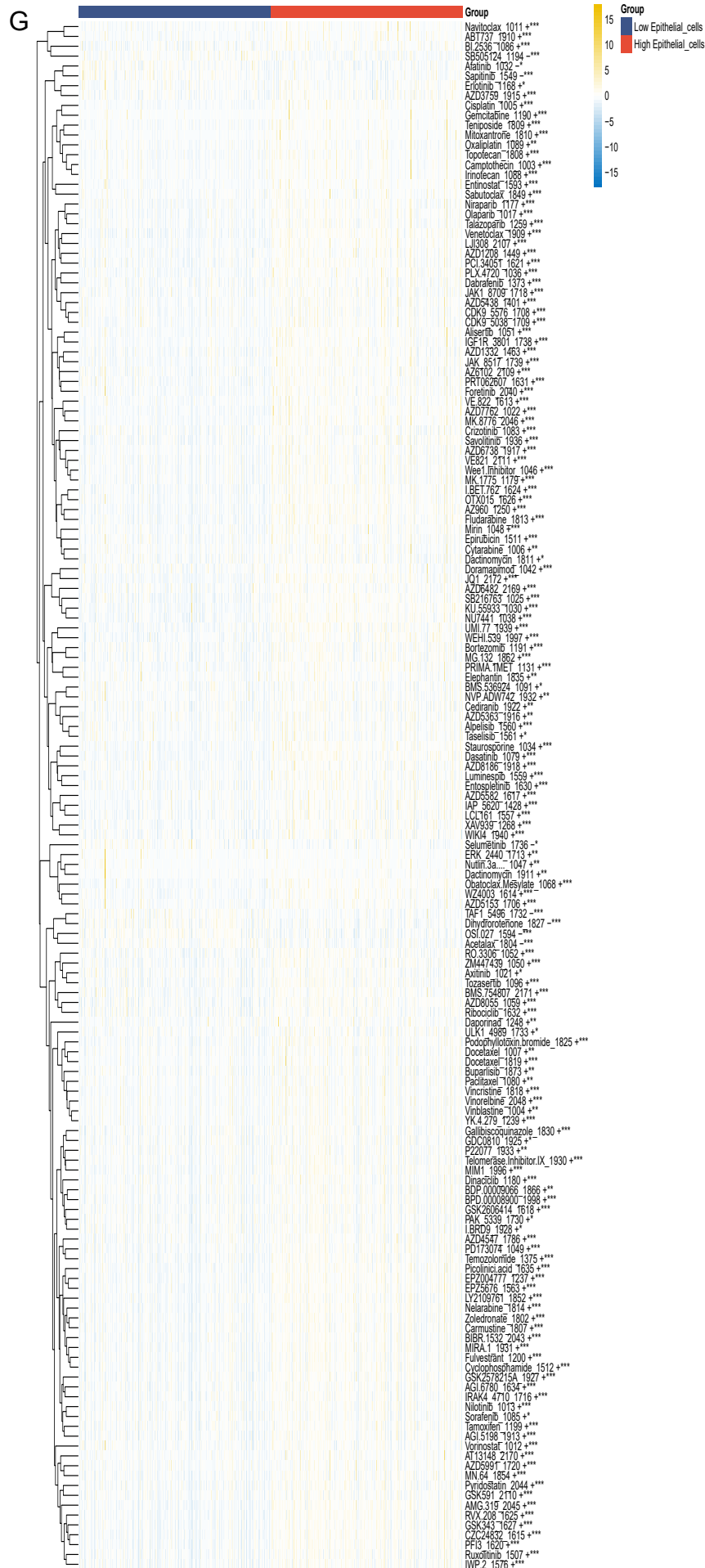

Supplement: Supplementary file 1 [file cdr-8-3-SupplementaryFiles.zip › cdr7091-SupplementaryFiles/Supplementary Figures/Supplementary Figure 8.pdf]

Response PR PD CR SD

Kruskal–Wallis,  $p = 0.0012$

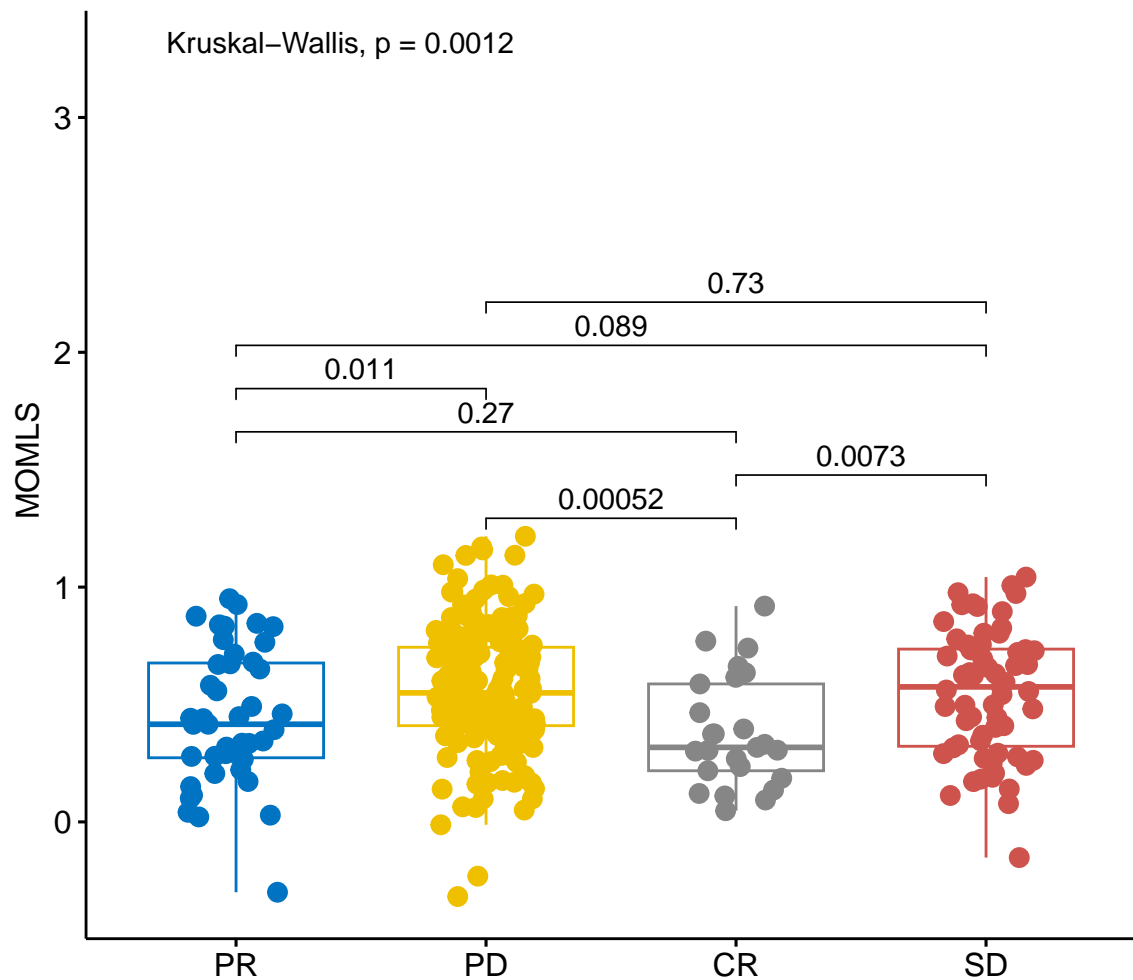

Supplement: Supplementary file 1 [file cdr-8-3-SupplementaryFiles.zip › cdr7091-SupplementaryFiles/Supplementary Figures/Supplementary Figure 9.pdf]
